# Novel *App* knock-in mouse model shows key features of amyloid pathology and reveals profound metabolic dysregulation of microglia

**DOI:** 10.1186/s13024-022-00547-7

**Published:** 2022-06-11

**Authors:** Dan Xia, Steve Lianoglou, Thomas Sandmann, Meredith Calvert, Jung H. Suh, Elliot Thomsen, Jason Dugas, Michelle E. Pizzo, Sarah L. DeVos, Timothy K. Earr, Chia-Ching Lin, Sonnet Davis, Connie Ha, Amy Wing-Sze Leung, Hoang Nguyen, Roni Chau, Ernie Yulyaningsih, Isabel Lopez, Hilda Solanoy, Shababa T. Masoud, Chun-chi Liang, Karin Lin, Giuseppe Astarita, Nathalie Khoury, Joy Yu Zuchero, Robert G. Thorne, Kevin Shen, Stephanie Miller, Jorge J. Palop, Dylan Garceau, Michael Sasner, Jennifer D. Whitesell, Julie A. Harris, Selina Hummel, Johannes Gnörich, Karin Wind, Lea Kunze, Artem Zatcepin, Matthias Brendel, Michael Willem, Christian Haass, Daniel Barnett, Till S. Zimmer, Anna G. Orr, Kimberly Scearce-Levie, Joseph W. Lewcock, Gilbert Di Paolo, Pascal E. Sanchez

**Affiliations:** 1 Denali Therapeutics, Inc., 161 Oyster Point Blvd, South San Francisco, California, 94080 USA; 2 Department of Pharmaceutics, University of Minnesota, 9-177 Weaver-Densford Hall, 308 Harvard St. SE, Minneapolis, MN 55455 USA; 3 Gladstone Institute of Neurological Disease, San Francisco, CA 94158 USA; 4 Department of Neurology, University of California, San Francisco, CA 94158 USA; 5 The Jackson Lab, Bar Harbor, Maine USA; 6 Allen Institute for Brain Science, Seattle, Washington, USA; 7 German Center for Neurodegenerative Diseases (DZNE) Munich, 81377 Munich, Germany; 8 Department of Nuclear Medicine, University Hospital of Munich, LMU Munich, Munich, Germany; 9 Metabolic Biochemistry, Biomedical Center (BMC), Faculty of Medicine, Ludwig- Maximilians-Universität, München, 81377 Munich, Germany; 10 Munich Cluster for Systems Neurology (SyNergy), 81377 Munich, Germany; 11 Appel Alzheimer’s Disease Research Institute, Weill Cornell Medicine, New York, NY USA; 12 Feil Family Brain and Mind Research Institute, Weill Cornell Medicine, New York, NY USA; 13 Neuroscience Graduate Program, Weill Cornell Medicine, New York, NY USA

**Keywords:** Neuritic plaques, Vascular amyloid, Neurodegeneration, Astrogliosis, Phagocytic microglia, Lipid dyshomeostasis

## Abstract

**Background:**

Genetic mutations underlying familial Alzheimer’s disease (AD) were identified decades ago, but the field is still in search of transformative therapies for patients. While mouse models based on overexpression of mutated transgenes have yielded key insights in mechanisms of disease, those models are subject to artifacts, including random genetic integration of the transgene, ectopic expression and non-physiological protein levels. The genetic engineering of novel mouse models using knock-in approaches addresses some of those limitations. With mounting evidence of the role played by microglia in AD, high-dimensional approaches to phenotype microglia in those models are critical to refine our understanding of the immune response in the brain.

**Methods:**

We engineered a novel *App* knock-in mouse model (*App*^SAA^) using homologous recombination to introduce three disease-causing coding mutations (Swedish, Arctic and Austrian) to the mouse *App* gene. Amyloid-β pathology, neurodegeneration, glial responses, brain metabolism and behavioral phenotypes were characterized in heterozygous and homozygous *App*^SAA^ mice at different ages in brain and/ or biofluids. Wild type littermate mice were used as experimental controls. We used *in situ* imaging technologies to define the whole-brain distribution of amyloid plaques and compare it to other AD mouse models and human brain pathology. To further explore the microglial response to AD relevant pathology, we isolated microglia with fibrillar Aβ content from the brain and performed transcriptomics and metabolomics analyses and *in vivo* brain imaging to measure energy metabolism and microglial response. Finally, we also characterized the mice in various behavioral assays.

**Results:**

Leveraging multi-omics approaches, we discovered profound alteration of diverse lipids and metabolites as well as an exacerbated disease-associated transcriptomic response in microglia with high intracellular Aβ content. The *App*^SAA^ knock-in mouse model recapitulates key pathological features of AD such as a progressive accumulation of parenchymal amyloid plaques and vascular amyloid deposits, altered astroglial and microglial responses and elevation of CSF markers of neurodegeneration. Those observations were associated with increased TSPO and FDG-PET brain signals and a hyperactivity phenotype as the animals aged.

**Discussion:**

Our findings demonstrate that fibrillar Aβ in microglia is associated with lipid dyshomeostasis consistent with lysosomal dysfunction and foam cell phenotypes as well as profound immuno-metabolic perturbations, opening new avenues to further investigate metabolic pathways at play in microglia responding to AD-relevant pathogenesis. The in-depth characterization of pathological hallmarks of AD in this novel and open-access mouse model should serve as a resource for the scientific community to investigate disease-relevant biology.

**Supplementary Information:**

The online version contains supplementary material available at 10.1186/s13024-022-00547-7.

## Background

Alzheimer’s disease (AD) is a devastating neurological condition. Research using preclinical AD models has been an essential step towards the development of therapies and discovery of disease-related biomarkers. In particular, mouse models recapitulating some aspects of this complex disease are instrumental in gaining insight into the neurobiology of AD [1]. Transgenic mouse models overexpressing human amyloid precursor protein (hAPP) with familial AD (fAD) mutations, sometimes in combination with *PSEN1* fAD mutations, have furthered our understanding of disease mechanisms [2–4], but also have the potential to introduce artifacts associated with *App* overexpression. Recently, a new generation of mouse models using knock-in (KI) approaches have been developed to avoid some of the limitations of transgenesis, including random genetic integration, ectopic expression and non-physiological protein levels [5]. In those models, the *App* gene has been humanized for the Aβ sequence and harbored fAD mutations resulting in progressive accumulation of amyloid deposits. Other pathologies associated with amyloid deposition have been observed, including immune responses and neurodegenerative processes [6, 7]. The identification of a plaque-induced gene signature (PIG) partially overlapping with gene expression changes observed in AD brain samples using an *App* KI model [8] further supports the relevance of these new models and provides incentive to continue their utilization to better define disease mechanisms and test candidate therapeutics.

Over the last decade, convergent findings from human genetics, gene expression studies and preclinical research revealed that microglia, the resident immune cells of the central nervous system (CNS), are likely contributing to AD pathophysiology [9–11]. Microglia respond to pathogenic drivers of AD, including amyloid-β (Aβ) and tau, but how microglia may become dysfunctional and contribute to disease remain unclear. Microglia expression profiles from AD and preclinical mouse models have revealed that microglia undergo profound changes in their gene expression profiles [8, 9, 12–19]. However, the functional implications of this molecular remodeling are poorly defined. It has been hypothesized that this gene expression program may enable microglia to sequester and/or resorb amyloid deposits and associated neurodegenerative processes. Under these circumstances, the phagocytic activity of microglia is critical and the metabolic demand and burden on microglial degradation pathways are high, potentially leading to immunometabolic alteration [20] and increasing cellular dysfunction over time [21–23]. Indeed, alterations of lipid metabolism have been observed in AD brain, and several genes associated with late onset AD (LOAD) risk that control lipid metabolism (*e.g.*, *TREM2*, *APOE*) are highly expressed in microglia [10, 24].

Here, we describe the engineering and characterization of a novel humanized Aβ *App* KI mouse model carrying the Swedish, Arctic and Austrian fAD mutations (*App*^SAA^). This model demonstrated elevated Aβ_42/40_ ratio in relevant tissues, progressive deposition of amyloid plaques, increased biomarkers of neuroinflammation and neurodegeneration, as well as mild behavioral phenotypes. Leveraging this new model, we discovered extensive lipid and metabolite alterations in microglia, associated with their level of phagocytic activity and proximity to amyloid plaques. Specifically, metabolic profiles of phagocytic microglia were consistent with lysosomal dysfunction (*e.g.*, accumulation of ganglioside GM3) and foam cell phenotypes (*e.g.*, accumulation of neutral lipids like triglycerides). Those findings were corroborated by gene expression changes in phagocytic microglia and highlight the profound reprogramming of microglia as they respond to amyloid pathology. Our high-dimensional profiling of microglia opens new avenues to explore the contribution of metabolic pathways in their response to AD pathology, including specific lipids and metabolites of interest.

## Methods

### Generation of *App*^SAA^ knock-in mouse model

The *App*^SAA^ knock-in mouse model was engineered by insertion of 6 mutations into the genomic *App* locus via homologous recombination. Three amino acids APP G676R, APP F681Y and APP R684H were substituted to humanize the mouse Aβ_1-42_ region and the following three FAD-linked APP mutations were inserted: KM670/671NL (Swedish), E693G (Arctic) and T714I (Austrian). This mouse model was created on a C57BL/6J background by Ozgene (Australia) using goGermline technology [25].

To generate the targeting vector for the *App*^SAA^ KI mouse model, six individual fragments (A-F) that cover the targeting area were engineered and introduced into six cloning vectors respectively. Fragment A encoding a 2828bp right homology arm fragment within intron 17 (mouse genomic DNA chr16:84,959,292-84,962,119 GRCm38/mm10) was amplified by PCR from BAC genomic DNA (clones RP23-126H12 and RP23-99P18) with primers P1915_41 and P1915_51. Fragment B encoding a portion of intron 17, exon 17 with the T714I and E693G mutations, a portion of intron 16 (corresponding to mouse genomic DNA region chr16:84,962,120-84,963,051 GRCm38/mm10) and a portion of the hygromycin cassette was synthesized as a gBlock by Integrated DNA Technologies. Fragment C encoding the remainder of the hygromycin cassette was amplified by PCR from an Ozgene in-house cloning vector using primers P1915_74 and P1915_53. Fragment D encoding a 2032bp portion of intron 16 (mouse genomic DNA region chr16:84,963,052-84,965,083 GRCm38/mm10) was synthesized as a gBlock by Integrated DNA Technologies. Fragment E encoding a portion of intron 16, exon 16 with the R684H, F681Y, G676R, and KM670/671NL mutations, and a portion of intron 15 (corresponding to mouse genomic DNA chr16:84,965,084-84,965,993 GRCm38/mm10) was synthesized by GENEWIZ and was housed within a vector that contained the neo cassette on the vector backbone. Fragment F encoding a 5870bp left homology arm within intron 15 (mouse genomic DNA chr16:84,965,994-84,971,863 GRCm38/mm10) was amplified by PCR from BAC genomic DNA (clones RP23-126H12 and RP23-99P18) with primers P1915_46 and P1915_56. The final targeting vector was then generated by the sequential assembly of fragments A-F. Fragment A digested with AatII was ligated into vector B digested with the same enzyme to generate vector AB. Fragment AB digested with AgeI was ligated into fragment C digested with the same enzyme to generate vector ABC. Fragment ABC digested with AscI was ligated into vector D digested with MluI to generate vector ABCD. Fragment ABCD digested with AscI was ligated into vector E digested with the same enzymes to generate vector ABCDE. Fragment ABCDE digested with enzyme MluI was ligated into vector F digested with the same enzyme to generate vector ABCDEF. The targeting vector containing Fragment ABCDEF was linearized with PmeI and electroporated into Bruce ES cells which were derived from C57BL/6-Thy1.1 mice. ES cells were maintained in the medium supplemented with G418 drug at 200ug/ml, and surviving clones were picked after 8 days of drug selection. ES cells were then screened for correct targeting events by qPCR and then confirmed by Southern analysis. For southern analysis, genomic DNA was purified from two ES clones (II_1C11 and II_1G11), digested with SpeI and then detected with 5’probe, 3’ probe, Hygro probe, enP probe, and neoP probe. Both clones were tested positive for proper homologous recombination in left and right arms. The ES cell clones (clone II_1C11 and clone II_1G11) that were confirmed to carry the correct homologous recombination events were injected into goGermline blastocysts, and the resulting chimeric mice were crossed to Flp transgenic mice (OzFlp) to excise the neo and hygro selection cassettes. The resulting heterozygous KI mice were backcrossed to C57BL/6J for more than two generations and then intercrossed to obtain homozygous KI mice for further characterization. The *App*^SAA^ model is available from the Jackson Laboratory as B6(Cg)-Apptm1.1Dnli/J (https://www.jax.org/strain/034711). Primer sequences and assays for mouse model generation are listed in the Supplementary Table 1.

### Animals

The *App*^SAA^ mice were maintained on the C57BL/6J genetic background. The *App*^SAA^ heterozygous KI mice were intercrossed to obtain three genotypes of interest. Other *App* transgenic lines were used for comparative analysis: 5xFAD (B6SJL-Tg(APPSwFlLon,PSEN1*M146L*L286V)6799Vas/Mmjax, MMRRC Strain #034840-JAX [26]), APP/PS1 (B6.Cg-Tg[APPswe, PSEN1dE9]85Dbo/Mmjax, MMRRC Strain #034832-JAX [27]), Tg2576 (B6;SJL-Tg[APPSWE]2576Kh a [28]). The *App*^SAA^ mice were bred either at the Jackson Laboratory (USA, Bar Harbor or Sacramento) or Ozgene (Australia, Perth). The 5xFAD mice were bred at the Jackson Laboratory (USA, Sacramento). The APP/PS1 and Tg2576 mice were studied at Allen Institute and all experimental procedures related to the use of these two lines of mice were approved by the Institutional Animal Care and Use Committee of the Allen Institute for Brain Science, in accordance with NIH guidelines. Before experiments, the *App*^SAA^ mice were shipped to Denali Therapeutics for an acclimation period of at least 2 weeks. Housing conditions were similar among labs and included standard pellet food and water *ad libitum*, a 12-h light–dark cycle at 22°C with a maximum of 5 mice per cage. *App*^SAA^ KI mice used for experiments are listed in the Supplementary Table 2. All mouse husbandry and experimental procedures were reviewed and approved by Denali Institutional Animal Care and Use Committee and were conducted in full compliance with regulatory statutes, Institutional Animal Care and Use Committee policies, and National Institutes of Health guidelines.

### Frailty index composite and voluntary running assays

The behavioral assays were conducted at the Neurobehavioral Phenotyping core of the Jackson Laboratory (Bar Harbor, USA). Mice were brought in the testing room at least 60 min before the assessment for habituation. For the Frailty Index composite assay, mice were weighed and evaluated one at a time in a battery of assessment. Except for a few quantitative measures (body weight and body temperature; Braintree Scientific #TH5 Thermalert), all other variables were scored in a scale of 0 to 1 by a trained technician. Characteristics observed include physical, physiological and innate reflex conditions (modified from Whitehead et al. [29]). To measure voluntary running, mice were individually placed in a new cage equipped with a running wheel (Med Associates) and the activity of the animals was recorded during a minimum of 2 days and 3 nights. Data were evaluated for onset of activity (typically correlated with lights off), distance traveled (rpm) and time spent running (min). To test the activity of the mice in a novel environment, mice were placed inside an open-field area (Omnitech Electronics, Columbus, OH, USA) for 60 min. The enclosure was surrounded by two levels of infrared photo beam sensors enabling recording of the horizontal and vertical activity of the mice.

### Repeated open field test

Exploratory locomotor activity was measured in an open field with a video-tracking system (EthoVision; Noldus) at the Gladstone Institute. 16–17-month-old *App*^SAA^ mice (KI/KI) (n=21, 11 males and 10 females) and wildtype littermate controls (+/+) (n=26, 14 males and 12 females) were used for the analysis. Mice were placed in one of four identical clear plastic circular chambers (12” diameter) for 5 min. The chamber walls were covered with plain gray backing to visually isolate the mice from their surroundings. Distance travelled in the center (0–4.5” diameter) and periphery (4.5–6” diameter) were measured in cm. Mice were tested twice a day (morning and afternoon sessions; 3–4 hours apart) on two consecutive days (Days 1–2; Trials 1–4) and retested two weeks later (Days 15–16; Trials 5–8) in the same chamber. The chambers were cleaned with 70% ethanol between trials. Only male or female mice were tested simultaneously in the four chambers, and to ensure balanced groups throughout the day, male and female mice were tested alternately. Statistical analyses were performed with SPSS (v.27).

### Tissue collection

Prior to tissue harvest, animals were deeply anesthetized via intraperitoneal (i.p.) injection of 2.5% Avertin. All collected tissues were snap-frozen on dry ice and stored at -80°C. Blood samples were collected by cardiac puncture into an EDTA tube (Sarstedt Microvette 500 K3E, Ref# 201341102), then slowly inverted 10 times prior to centrifugation at 12,700 rpm for 7 minutes at 4°C to collect plasma. CSF samples were collected by pre-pulled glass capillary tube from the cisterna magna and then transferred to 0.5 mL Protein LoBind Eppendorf tubes (Eppendorf Cat #022431064) for centrifugation at 12,700 rpm for 7 minutes at 4°C, supernatant was then collected. The mice were then perfused intracardially with cold PBS, and brain tissues were sub-dissected to separate the cortical and hippocampal regions.

### SDS-PAGE and Western blotting

Brains were homogenized using Qiagen Tissue-Lyser II (28 Hz, 3 min, 3 times) in lysis buffer (Cell Signaling #9803) containing protease inhibitor cocktail (Roche #4693159001) and PhosSTOP (Roche #4906837001) (1 ml lysis buffer for 100 mg tissue). Lysates were then incubated on ice for 10 min followed by centrifugation at 18,660 g for 20 min at 4°. Supernatants were transferred to new tubes for protein concentration determination and loading sample preparation. Protein lysate samples were boiled at 95°C and SDS-PAGE was performed using standard BioRad reagents. For Western blotting, PDVF membranes were incubated overnight at 4°C with the following primary antibodies diluted blocking buffer (Rockland): Mouse anti APP A4 clone 22C11 (Millipore MAB348, 1:1,000), Mouse anti APP clone 6E10 (Biolegend 803001, 1:2,000), Rabbit anti APP CTF (Sigma A8717, 1:4,000), Mouse anti-β-Actin (Sigma A2228, 1:4,000). Membranes were then incubated with the appropriate fluorescently conjugated secondary antibody (1:10,000, Li-Cor) and imaged using a Li-Cor Odyssey CLx system.

### Aβ extraction and measurement by MSD

Cortical and hippocampal tissues were weighed and homogenized in TBS (140 mM NaCl, 3 mM KCl, 25 mM Tris-HCl, pH 7.4, 5 mM EDTA, 2 mM 1,10-phenanthroline) containing protease inhibitor (Roche,#4693159001). The homogenates were centrifuged at 100,000 g, 4°C for 1 hour, and then the supernatant was collected (TBS-soluble fraction). Pellets were resuspended in 5 M guanidine, 50 m M Tris, pH 8.0, further homogenized, and incubated at room temperature for 3 hours. Samples were then centrifuged at 20,800 g, 4°C, for 20 minutes, and the supernatant was collected (TBS-insoluble GuHCl fraction). To measure Aβ in brain fractions and biofluids, we tested two commercial kits: MSD V-PLEX Aβ Peptide Panel 1 (4G8) Kit (cat#K15199E) and MSD V-PLEX Aβ Peptide Panel 1 (6E10) Kit (cat#K15200E). We found that the 4G8 kit has a good cross-reactivity to mouse and human Aβ. Since the 4G8 kit showed a greater sensitivity than the 6E10 kit to detect mutated human Aβ from 4-month *App*^SAA^ KI/KI brains, we used 4G8 kit for Aβ analysis. Samples were diluted by Diluent 35 provided in the kit (GuHCl fractions 1:20; TBS fractions, no dilution; plasma 1:4; CSF 1:26). Plates were read using the SECTOR Imager 2400A.

### Tau measurement by MSD

Total mouse tau in CSF was measured using an ECL-immunoassay MSD 96-well mouse total tau assay (Meso Scale Discovery: MSD) according to the manufacturer’s instructions. Briefly, CSF samples (5 μL) were diluted 1 to 5 in 10% blocking solution (10% BSA in tris wash buffer) and incubated at room temperature for 1 hour on a shaker plate. Standard samples were also diluted in 10% blocking solution. Plates were washed 4 times with 1XTris washing buffer and then incubated at room temperature with sulfo-tag total tau detection antibody for 1 hour. After a few additional washes, plates were read on the MSD SECTOR 600 Reader. Samples were fit against a 9-point standard curve.

### TREM2 measurement by MSD

TREM2 levels in brain homogenates prepared by Cell Signaling lysis buffer (#9803) and plasma were quantified by using MSD technology. The sandwich layout of this TREM2 MSD assay from the bottom to the top is (1) capture-antibody (1 μg/mL; BAF1729, R&D Systems), (2) samples, (3) primary-antibody (10 μg/mL; anti-TREM2 antibody - clone 4D9) [30], and (4) detection-antibody (0.5 μg/mL; SULFO-TAG-labeled anti-human antibody, Meso Scale Discovery). MSD 96-well plates coated with streptavidin were first incubated with 150 μL MSD-Blocker A. For building each layer for the assay, 25 μL of the reagent was added to each well, and the plates were incubated at room temperature on a shaker at 800 rpm for 1hr. Between incubation step, each well was washed with TBST buffer (0.05% Tween 20 in TBS) using ELx406 plate washer (BioTek). Diluted samples (Brain lysates = 1:5; plasma = 1:20) and TREM2 protein standard in assay buffer (25% MSD-Blocker A and 75% TBST) were prepared and aliquoted for running duplicates in the assay. A four-fold serial-dilution standard curve of recombinant murine TREM2 extracellular domain was prepared to include the linear detection range from 62.5 ng/ml to 15.25 pg/mL. For acquiring the MSD units on the MSD Sector Imager S600 reader (MSD), 150μL of 2X MSD read buffer was added to each well after the final TBST wash. TREM2 levels were calculated using the MSD Discovery Workbench software and normalized by each lysate's concentration (brain samples only).

### Cytokines

Brain homogenates prepared by Cell Signaling lysis buffer (#9803) were diluted to 5 μg/μl with PBS and plasma samples were prepared at a 2-fold dilution with PBS. Diluted samples were sent to Eve Technologies (Canada) for cytokines and chemokines measurement by using the Mouse Cytokine Array / Chemokine Array 44-Plex (MD44).

### Neurofilament Light detection

CSF neurofilament light (Nf-L) concentrations were measured using Simoa NF-Light® (SR-X version, Quanterix 103400) bead-based digital ELISA kits and read on the Quanterix SR-X instrument. CSF samples were diluted 100x with Sample Diluent (Quanterix 102252) before being added to Simoa 96-well microplates (Quanterix 101457). Following kit instructions, Simoa Detector Reagent and Bead Reagent (Quanterix 103159, 102246) were added to the samples before incubating and shaking for 30 mins, 30°C at 800 rpm. After incubation, the sample plate was washed with Simoa Wash Buffer A (Quanterix 103078) on a Simoa Microplate Washer according to Quanterix’s two step protocol. After initial washes, SBG Reagent (Quanterix 102250) was added and samples were again incubated at 30°C, 800 rpm for 10 min. The two-step washer protocol was continued, with the sample beads being twice resuspended in Simoa Wash Buffer B (Quanterix 103079) before final aspiration of buffer. Sample Nf-L levels were measured using the NF Light analysis protocol on the Quanterix SR-X instrument and interpolated against a calibration curve provided with the Quanterix assay kit.

### Whole brain imaging and quantification of plaque distribution

To label amyloid plaques, mice were administered 3.3 mg/kg of methoxy-X04 (R&D Systems) by intraperitoneal (i.p.) injection. Twenty-four hours later, mice were perfused with PBS and 4% paraformaldehyde (PFA) sequentially and then intact brains were dissected and postfixed in 4% PFA at room temperature for 6 hours, followed by overnight at 4°C. Whole brain fluorescence imaging was performed as described in Whitesell et a l[31] with serial two-photon (STP) tomography (TissueCyte 1,000, TissueVision Inc., Somerville, MA), using 925 nm excitation, a 500 nm dichroic mirror, and a 447/60 bandpass emission filter on the blue channel. One hundred and forty serial block-face images were acquired from each brain at 0.35 μm/pixel lateral resolution with a 100 μm sectioning interval. Automated segmentation of the fluorescent signal from methoxy-X04 labeled plaques and registration to the 3D Allen Mouse Brain Common Coordinate Framework, v3 (CCFv3) [32] were performed as previously described [31]. Briefly, segmented fluorescence output is a full resolution mask that classifies each 0.35 μm × 0.35 μm pixel as either signal or background. An isotropic 3D summary of each brain is constructed by dividing each image series into 10 μm × 10 μm × 10 μm grid voxels. Total signal is computed for each voxel by summing the number of signal positive pixels in that voxel. Each image stack is registered to the CCFv3 in a multi-step process using both global affine and local deformable registration. Plaque density for each structure in the reference atlas ontology was calculated by summing voxels from the same structure. To obtain plaque counts within each structure, we used a standard feature labeling algorithm. Adjacent and orthogonally adjacent voxels in the segmentation signal were grouped together as one plaque object. Due to the 100 μm z-sampling interval, our resolution limit for detecting separate plaques in the z-axis was 100 μm. Plaque quantification is reported for one hemisphere per brain, chosen based on image and tissue quality (Supplementary Table 3). All image series were subjected to manual QC checks for completeness and uniformity of raw fluorescence images, minimum fluorescence intensity, and artifacts. Automatic segmentation results were checked for overall quality and false positive signals by overlaying segmentation results for 3–5 single coronal sections with raw fluorescent images from STP imaging.

### Sectioning and immunofluorescence staining

Fresh brains were fixed by immersion in 4% paraformaldehyde at 4°C for 24 hours then transferred to a phosphate buffered saline (PBS) solution with 0.1% sodium azide for storage until ready for processing. Brains were initially transferred to a 30% sucrose solution in PBS for two days before sectioning and then subsequently sectioned coronally on a freezing microtome at a thickness of 40 μm for most stainings except for astrocyte-related stainings (GFAP, GLT-1 and C3), which were performed on 30 μm sections. Sections were stored in cryoprotectant buffer (30% glycerol, 30% ethoxyethanol, and 40% PBS) at -20°C prior to staining. Seven to twelve coronal brain sections (from approximately bregma to 4.8 mm posterior to bregma) were selected for immunostaining for Aβ, Iba1, CD68, AT8, LAMP1, Neurofilament, CD31, alpha smooth muscle actin, GFAP, GLT-1 and C3. Sections were incubated for 1 hour at room temperature in 1x Tris-buffered saline solution containing 0.05% Tween (TBST) and 5% donkey serum, followed by primary antibodies overnight at 4°C. Sections were then washed in TBST and a solution of secondary antibodies was then applied for 1 hour at room temperature. Sections were washed in TBST prior to mounting and cover slipping with Prolong Glass Antifade Mountant solution (Thermo Fisher, P36984). Immunofluorescence was performed using the following primary antibodies: rabbit anti-human amyloid beta (IBL America, 18584; 1:500), rat anti-CD68 (BioRad, MCA1957A; 1:500), goat anti-Iba1 (Novus, NB100-1028; 1:500), mouse anti-pTau (Invitrogen, MN1020; 1:500), goat-anti-CD31 (R&D Systems, AF3628, 1:500), mouse-anti-alpha smooth muscle actin Cy3 (Sigma, C6198; 1:500), rat anti-LAMP1 (DSHB, 1D4B; 1:250), mouse anti-Neurofilament (BioLegend, 837801; 1:500), rabbit anti-GFAP (Sigma, G9269, 1:500), guinea pig anti-GLT-1 (Sigma, AB1783, 1:1000) and rat anti-complement component 3 (C3; clone 11H9, Novus Biologicals, NB200-540, 1:50); and the following secondary antibodies: Alexa Fluor 488 Donkey anti-rabbit IgG (Thermo Fisher, A-21206; 1:200), DyLight 550 Donkey anti rat-IgG (Thermo Fisher, SA5-10027, 1:200), Alexa Fluor Plus 555 Donkey anti-mouse IgG (Invitrogen, A32773; 1:200), Alexa Fluor 647 Donkey anti-goat IgG (Thermo Fisher, A-21447; 1:200), Alexa Fluor Plus 647 Donkey anti-mouse IgG (Thermo Fisher, A32787; 1:200), Alexa Fluor 488 donkey anti-mouse IgG (Thermo Fisher, A21202; 1:500), Alexa Fluor 555 goat anti-rat IgG (Thermo Fisher, A21434; 1:500), Alexa Fluor 555 goat anti-guinea pig IgG (Thermo Fisher, A21435; 1:500), Alexa Fluor 555 donkey anti-rabbit IgG (Thermo Fisher, A31572; 1:500).

### Multiplexed Fluorescence in situ hybridization and imaging

Fresh-frozen mouse brains embedded in OCT were coronally sectioned at 15 μm onto Superfrost Plus glass slides (Fisher Scientific). Sections were stored at -80°C until RNAscope treatment. The RNAscope multiplex fluorescent v2 kit was used following the manufacturer’s protocol for fresh-frozen tissue sections (ACD 323100) except that protease treatment was performed for 15 minutes. Probe sets for *Trem2* (ACD 404111-C2) and *Tmem119* (ACD 472901-C3) were used with Opal 520 and Opal 570 dyes (Akoya Biosciences FP1487001KT), respectively. Methoxy-X04 (Tocris 4920) was applied to the tissue sections at 100 μM in 1xPBS for 30 min at room temperature immediately following *in situ* hybridization. Sections were then washed three times for 10min in 1xPBS before mounting in ProLong Gold Antifade Mountant (ThermoFisher P36930) followed by a coverslip. Sections were imaged using a confocal microscope (Leica SP8; Leica Microsystems, Inc.) with a 20x/.75IMM oil objective.

### Microscopy and quantification for immunohistochemistry

Fluorescent stained sections were captured using a Zeiss Axioscan.Z1 slide scanner with a 10x / 0.45 NA objective. A custom macro in Zeiss Zen software was used to quantify signal for Aβ, CD68, Iba1 and AT8 following median smoothing, channel extraction, and local background subtraction to create binary masks of fluorescent signal for each channel. Objects smaller than 10 pixels were excluded as staining artifacts / debris. Individual plaques were dilated to create a region surrounding the plaques to detect microglia adjacent to plaques. The cortex and hippocampus regions of interest (ROIs) were manually outlined for each section and analyzed using the macro to quantify ROI area and measure the number and area of all plaques and microglia, including overlapping area between microglia and the dilated region surrounding and including plaques. The percentage amyloid plaque, Iba1, CD68, or AT8 positivity in the areas of interest were calculated by normalizing the positive pixel area to the quantified tissue area and averaged from 7–12 sections per mouse.

For 3D assessment of plaque architecture and the associated cellular changes, super resolution confocal microscopy of fluorescently stained sections was performed using a scanning confocal microscope (Leica SP8, Leica Microsystems, Inc.) operated in super resolution lightning mode. Images were acquired using a 63x / 1.4 NA oil immersion objective at a pixel size of 50 nm and processed using the Adaptive processing algorithm. Confocal z-stacks of 18-25 μm were acquired for each channel using sequential scan settings. A minimum of four representative fields were captured from within the cortical brain region of *App*^SAA^ KI/KI (n=3-4) and *App*^SAA^ +/+ control (n=2) mice.

Microscopic evaluation of cerebral amyloid angiopathy (CAA) pathology was performed on larger fields using a 10x / 0.45 NA objective at a pixel size of 150 nm and z-stacks of 30-40 μm, and at higher resolution using a 20x / 0.75 NA oil immersion objective at a pixel size of 67 μm and z-stacks of 25-35 μm. In both cases, images were processed using the lightning Adaptive processing algorithm.

To assess the 3D morphology of microglia and sub-cellular localization of methoxy-X04 and Aβ, a minimum of 3 cortical regions per animal were imaged in *App*^SAA^ KI/KI mice (n=6) using a 40x/1.3 NA oil immersion objective at a pixel size of 50 nm in Lightning mode and processed using the Adaptive processing algorithm. Confocal z-stacks of 15-30 μm with a z-step size of 500 nm were acquired for each channel using sequential scan settings. To reduce noise, images were first resampled to 500 nm isotropic volumes using local mean resampling. The background intensity of each image was estimated using gaussian smoothing with a kernel size of 10 μm isotropic and subtracted from the original image. The methoxy-X04 and Aβ surfaces were estimated by manual thresholding of each image. The surface of Iba1+ microglia was estimated using a bipartite manual threshold, where values below the lowest value were assigned background, values above the high threshold were assigned to the microglial volume, and intermediate values were assigned using random walker diffusive segmentation algorithm with a diffusion constant of 10 [33]. Microglia surfaces were found to often contain several microglia, so the positions of individual microglial soma were then estimated by finding regions where the surface was at least 7 μm in diameter, spaced at least 10 μm from other regions of high surface thickness, and then finding local peak cell thickness using non-maximum suppression. These regions of peak thickness were then used as seeds for a watershed segmentation of the overall microglial surface, where each voxel in the surface was assigned to the closest peak. Both segmentation stages were performed using the algorithms implemented in the Python package scikit-image v0.18.1 [34].

To assess astrogliosis, image acquisition of entire hemispheres, hippocampal subregions, and posterior parietal cortex was performed using the BX-X710 microscope (Keyence) with 4X, 20X or 40X objectives (Nikon). Image analysis was performed with FIJI [35]. Briefly, for GFAP immunoreactivity, images were background-subtracted and thresholded to clearly outline astrocytic cells and the total GFAP-positive area was measured. For C3 immunoreactivity, a GFAP-positive mask was generated and C3 fluorescence intensity was measured in GFAP-positive astrocytes. For GLT-1 immunoreactivity, total fluorescence intensity was measured. Statistical analysis was performed using GraphPad Prism (v9.3.1).

### Fluorescence activated cell sorting (FACS)

Mice were perfused with cold PBS and cortical and hippocampal tissues were dissected and dissociated into a single cell suspension using the Adult Brain Dissociation Kit (Miltenyi Biotec 130-107-677), according to the manufacturer’s protocol. Cell suspensions were stained with the following antibodies in FACS buffer (1% fatty acid-free BSA + 1mM EDTA in PBS) for 25 minutes on ice: Fixable Viability Stain BV510 (BD Biosciences, 564406), CD11b-BV421 (BioLegend 101251), CD45-APC (BD Biosciences, 559864), ACSA-2-PE (Miltenyi Biotec, 130-102-365), Mouse Fc blocker (anti-mouse CD16/32, BioLegend 101320). Cells were washed twice with FACS buffer and strained through a 100 μm filter before sorting CD11b^+^ microglia and ACSA-2^+^ astrocytes on a FACS Aria III (BD Biosciences) with a 100 μm nozzle. For bulk RNAseq analysis, cells were sorted directly into freshly prepared RLT-plus buffer (Qiagen) containing beta-mercaptoethanol; for lipid extraction, cells were sorted directly into LC/MS grade Methanol with internal standards.

To label Aβ-containing microglia, mice were injected i.p. with 10 mg/kg of methoxy-X04 (R&D Systems). Mice were perfused with PBS 24 hours after injection and cortical and hippocampal tissues were dissected and processed into single-cell suspension for staining as described above except the antibodies used for FACS were: Fixable/viability Dye 780 (Thermo fisher 65-0865-14), Cd11b-PE (BioLegend, 101208), CD45-APC (BD Biosciences, 559864), Mouse FC block 1:50 (BioLegend, 101320). Methoxy-X04 positive and negative microglia (CD11b+) were collected for RNAseq and LC-MS analysis.

### RNA isolation, RT-qPCR, and SMARTSeq library preparation

RNA from bulk mouse brain tissue was extracted using the RNeasy Plus Mini Kit (Qiagen) and resuspended in nuclease-free water. For RT-qPCR, 3 μl of RNA was transcribed into cDNA using SuperScript IV (Invitrogen). *App* gene expression level was assessed using Taqman probe (FAM-Mm01344172_m1) on a QuantStudio 6 Flex (Applied Biosystems) and normalized to *Gapdh*.

For RNA-seq analysis of FACS sorted cells, 200 live cells were sorted directly into 11.5 microliters of Clontech SMARTseq 10X reaction buffer for reverse transcription. cDNA synthesis was performed with the Clontech SMARTSeq v4 3′ DE kit (Takara Bio USA, Inc. 635040). Reverse transcription was followed by 14 cycles of cDNA amplification. cDNA was purified with 0.8X volume of SPRIselect beads (Beckman Coulter B23318), quantified on a Bioanalyzer 2100 System with a High Sensitivity DNA chip (Agilent 5067-4626). 300 pg of cDNA from each sample was used as input for library preparation with the Nextera XT DNA Library Prep Kit (Illumina FC-131-1096). Fragmentation and adaptors insertion were performed by tagmentation, followed by 12 cycles of PCR amplification. The final libraries were purified using 0.8X SPRIselect beads. Library quantity and quality were assessed with Qubit 4 Fluorometer with the 1X dsDNA HS Assay Kit (Invitrogen Q33231) and on a Bioanalyzer 2100 System with a High Sensitivity DNA chip. Sequencing reads were generated on an Illumina NovaSeq 6000 instrument (100 bp single end) by SeqMatic (Fremont, CA, USA).

Sequencing adapters were trimmed from the raw reads with skewer (version 0.2.2) [36]. Reads were aligned to the mouse genome version GRCm38_p6. A STAR index (version 2.7.1a) [37] and built with the --sjdbOverhang=50 argument. Splice junctions from Gencode gene models (release M17) were provided via the --sjdbGTFfile argument. STAR alignments were generated with the following parameters: --outFilterType BySJout, --quantMode TranscriptomeSAM, --outFilterIntronMotifs RemoveNoncanonicalUnannotated, --outSAMstrandField intronMotif, --outSAMattributes NH HI AS nM MD XS and --outSAMunmapped Within. Alignments were obtained with the following parameters: --readFilesCommand zcat --outFilterType BySJout --outFilterMultimapNmax 20 --alignSJoverhangMin 8 --alignSJDBoverhangMin 1 --outFilterMismatchNmax 999 --outFilterMismatchNoverLmax 0.6 --alignIntronMin 20 --alignIntronMax 1000000 --alignMatesGapMax 1000000 --quantMode GeneCounts --outSAMunmapped Within --outSAMattributes NH HI AS nM MD XS --outSAMstrandField intronMotif --outSAMtype BAM SortedByCoordinate --outBAMcompression 6. Gene level counts were obtained using featureCounts from the subread package (version 1.6.2 )[38] . Gene symbols and biotype information were extracted from the Gencode GTF file.

### RNA-seq data analysis

Following alignment and expression quantitation, lowly expressed genes were removed, and differential expression analysis was performed using the limma/voom with sample weighting framework [39, 40]. Lowly expressed genes are those that did not have more than ten reads assigned to them in at least as many samples as the minimum replicate size per experiment (six samples in GSE158152 reporting on total microglia analysis and ten in GSE158153 reporting on methoxy-X04 (+) vs methoxy-X04 (-) microglia). Technical replicate libraries from GSE158152 (identified by the *animal_id* column) were summed together prior to analysis. Linear models were fit against the covariate(s) of interest (genotype and/or methoxy-X04-status) with “take-down day” and “sex” encoded as batch covariates. Because the direct comparison of the methoxy-X04 (+) vs methoxy-X04 (-) profiles in *App*^SAA^ KI/KI mice utilized repeated measures from the same animal, “subject_id” was fit as a random effect using the duplicateCorrelation function from limma [41]. Empirical Bayes moderated t-statistics and *p-*values were computed relative to a 1.2 fold-change cutoff using treat [42]. Significantly differentially expressed genes were then defined as those having an FDR <= 10%.

Single sample gene set activity scores were calculated in Fig. 3b by taking a weighted average of the expression of the genes in each gene set using the eigenWeightedMean function from the sparrow Bioconductor package (v1.0.1; DOI: 10.18129/B9.bioc.sparrow). Weights correspond to the loadings of each gene on the first principal component of the mean-centered gene-by-sample matrix for each gene set. Activity scores for 5xFAD mouse profiles were taken from the microarray data reported in Ulland et al. [43] (GSE65067).

Gene set enrichment analyses were performed via the fgseaMultiLevel function in the fgsea R package using the moderated t-statistic as the gene ranking statistic [44]. Gene sets used for testing were taken from the Biological Process collection of the Gene Ontology database, the KEGG database, as well as a custom set of genes compiled from the literature that are enumerated in Supplemental Table 4 [45–47]. Gene set enrichment scores shown Fig. 4i were calculated by averaging the moderated t-statistics of the genes in the leading edge of the gene set. Because the leading edge can consist of different genes for each comparison, we defined the genes used in this calculation as the universe of the genes that appeared in the leading edge of the gene set across all comparisons shown in these figures. The gene sets presented in Fig. 4i along with the genes that make up their leading edge are listed in Supplementary Table 4. The methoxy-X04 (+) specific gene signature was extracted from Supplementary Table 4 (Specific X04+ DEGs) from Grubman et al. [13] by selecting only those genes with an estimated log2FC >= 1.

All software versions for the RNA-seq analysis correspond to Bioconductor release version 3.14 [48].

### FACS lipid extraction

Lipid extraction was performed using Matyash liquid-liquid extraction protocol with the following modifications. Briefly, 50,000 cells were sorted directly into 400 μl of methanol containing surrogate internal standards and kept on ice. To each tube, 200-400 μl of water was added to achieve final volume of 800 μl. These samples were vortexed for 5 min at room temperature. To these samples, 800 μl of tert-Butyl methyl ether (MTBE) was added and vortexed for an additional 5 min at room temperature. Samples were then centrifuged at 21,000 x g for 10 min at 4°C. Following centrifugation, 700 μl of upper organic layer was collected and dried under constant stream of N_2_ gas. Dried samples were reconstituted in 100 μl of MS-grade methanol for further analysis by LC-MS/MS.

### LCMS analysis of lipids

Lipid analyses were performed by liquid chromatography UHPLC Nexera X2, coupled to electrospray mass spectrometry (QTRAP 6500+, Sciex). For each analysis, 5 μL of sample was injected on a BEH C18 1.7 μm, 2.1×100 mm column (Waters) using a flow rate of 0.25 mL/min at 55°C. Electrospray ionization was performed in positive and negative ion modes. For positive ionization mode, mobile phase A consisted of 60:40 acetonitrile/water (v/v) with 10 mM ammonium formate + 0.1% formic acid; mobile phase B consisted of 90:10 isopropyl alcohol/acetonitrile (v/v) with 10 mM ammonium formate + 0.1% formic acid. For negative ionization mode, mobile phase A consisted of 60:40 acetonitrile/water (v/v) with 10 mM ammonium acetate + 0.1% acetic acid; mobile phase B consisted of 90:10 isopropyl alcohol/acetonitrile (v/v) with 10 mM ammonium acetate + 0.1% acetic acid. The gradient was programmed as follows: 0.0-8.0 min from 45% B to 99% B, 8.0-9.0 min at 99% B, 9.0-9.1 min to 45% B, and 9.1-10.0 min at 45% B.

Electrospray ionization was performed using the following settings: curtain gas at 30 psi; collision gas at 8 psi; ion spray voltage at 5500 V (positive mode) or -4500 V (negative mode); temperature at 250°C (positive mode) or 600°C (negative mode); ion source Gas 1 at 55 psi; ion source Gas 2 at 60 psi; entrance potential at 10 V (positive mode) or -10 V (negative mode); and collision cell exit potential at 12.5 V (positive mode) or -15.0 V (negative mode). Data acquisition was performed in multiple reaction monitoring mode (MRM) with the collision energy (CE) values reported in Supplementary Table 5. Lipids were quantified as area normalized to specific non-endogenous internal standards as reported in Supplementary Table 5. Quantification was performed using MultiQuant 3.02 (Sciex). Lipids were normalized to protein amount. Protein concentration was measured using the bicinchoninic acid (BCA) assay (Pierce, Rockford, IL, USA).

### LCMS analysis of polar metabolites

#### Positive mode

Metabolites analyses were performed liquid chromatography (UHPLC Nexera X2) coupled to electrospray mass spectrometry (QTRAP 6500+, Sciex). For each analysis, 5 μL of sample was injected on a BEH amide 1.7 μm, 2.1×150 mm column (Waters Corporation, Milford, Massachusetts, USA) using a flow rate of 0.40 mL/min at 40°C. Mobile phase A consisted of water with 10 mM ammonium formate + 0.1% formic acid. Mobile phase B consisted of acetonitrile with 0.1% formic acid. The gradient was programmed as follows: 0.0–1.0 min at 95% B; 1.0–7.0 min to 50% B; 7.0–7.1 min to 95% B; and 7.1–10.0 min at 95% B. The following source settings were applied: curtain gas at 30 psi; collision gas was set at at 8 psi; ion spray voltage at 5500 V; temperature at 600°C; ion source Gas 1 at 50 psi; ion source Gas 2 at 60 psi; entrance potential at 10 V; and collision cell exit potential at 12.5 V. Data acquisition was performed in multiple reaction monitoring mode (MRM) with the collision energy (CE) values reported in Supplementary Table 6. Quantification was performed using MultiQuant 3.02 (Sciex).

#### Negative mode

Metabolites analyses were performed liquid chromatography (ACQUITY I-Class Plus UPLC FL, Waters Corp) coupled to electrospray mass spectrometry (XEVO TQ-S Micro, Waters Corp). For each analysis, 5 μL of sample was injected on an Agilent InfinityLab Poroshell 120 HILIC-Z P 2.7 μm, 2.1×50 mm (Agilent Technologies Inc., Santa Clara, CA USA); using a flow rate of 0.5 mL/min at 25°C. Mobile phase A consisted of water with 10 mM ammonium acetate + 5 μM medronic acid (Agilent), pH 9. Mobile phase B consisted of acetonitrile:water 9:1 with 10 mM ammonium acetate + 5 μM medronic acid (Agilent), pH 9. The gradient was programmed as follows: 0.0–1.0 min at 95% B; 1.0–6.0 min to 50% B; 6.0–6.5 min to 95% B; and 6.5–10.0 min at 95% B. Electrospray ionization was performed in negative ion mode. The following source settings were applied: capillary voltage at 1.9 kV; source temperature at 150°C; desolvation temperature at 600°C; desolvation gas flow at 1000 L/hr; cone gas flow at 50 L/hr; cone voltage at 20 V; nebulizer gas at 7 bar. Data acquisition was performed in multiple reaction monitoring mode (MRM) with the collision energy (CE) values reported in Supplementary Table 6. Quantification was performed using Skyline (v19.1;University of Washington).

### In vivo PET imaging and quantification

All rodent PET procedures followed an established standardized protocol for radiochemistry, acquisition times and post-processing [49], which was transferred to a novel PET/MRI system. In brief, [^18^F]GE-180 TSPO-PET and [^18^F]FDG-PET was used to measure cerebral microglial activity and glucose metabolism respectively. For TSPO-PET imaging, *App*^SAA^ mice (KI/KI) (n=21, 14 males and 7 females) and aged-matched wildtype littermate controls (+/+) (n=30, 6 males and 24 females) were analyzed from 5 to 20 months of age. For FDG-PET imaging, another cohort of *App*^SAA^ mice (KI/KI) (n=14, 7 males and 7 females) and aged-matched wildtype littermate controls (+/+) (n=27, 6 males and 21 females) were studied from 5 to 20 months of age.

All mice were scanned with a 3T Mediso nanoScan PET/MR scanner (Mediso Ltd, Hungary) with a triple-mouse imaging chamber. A 15-minute anatomical T1 MR scan was performed at 15 min after [^18^F]FDG injection or at 45 min after [^18^F]GE-180 injection (head receive coil, matrix size 96 × 96 × 22, voxel size 0.24 × 0.24 × 0.80 mm^3^, repetition time 677 ms, echo time 28.56 ms, flip angle 90°). PET emission was recorded at 30-60 min p.i. ([^18^F] FDG) or at 60-90 min p.i. ([^18^F]GE-180). PET list-mode data within 400-600 keV energy window were reconstructed using a 3D iterative algorithm (Tera-Tomo 3D, Mediso Ltd, Hungary) with the following parameters: matrix size 55 × 62 × 187 mm^3^, voxel size 0.3 × 0.3 × 0.3 mm^3^, 8 iterations, 6 subsets. Decay, random, and attenuation correction were applied. The T1 image was used to create a body-air material map for the attenuation correction.

Normalization of injected activity was performed by the previously validated myocardium correction method [50] for [^18^F]GE-180 TSPO-PET and by standardized uptake value (SUV) normalization for [^18^F]FDG-PET.

FDG-PET and TSPO-PET values deriving from a predefined cortical volume of interest [51] were extracted and analyzed as a function of age in direct comparison of *App*^SAA^ and wildtype mice.

### Statistical analysis

Data have either been expressed as means ± SEM or as indicated in graphs. Statistical analysis of data was performed in GraphPad Prism 8 or as indicated otherwise for whole brain plaque analysis, RNAseq and LC-MS data. Analysis was performed using *t* test or with one-way analysis of variance (ANOVA) with Dunnett multiple comparison as indicated in figure legends. Criterion for differences to be considered significant was *P* < 0.05.

Comparison of LC/MS results from FACS-isolated microglia from *App*^SAA^ +/+, *App*^SAA^ KI/+ and *App*^SAA^ KI/KI mice: Integrated peak areas were divided by the area of pre-assigned internal limma standards, and the resulting ratios were log2 transformed. Analytes detected in less than 75% of the samples were removed. To identify and account for unwanted sources of variation, two surrogate variables (SV) were identified with the sva R packag e[52]. Genotypes were compared by fitting the following linear model using the limma R packag e[53]: log_2_(abundance) ~ genotype + batch + sex + SV. Sample weights were incorporated vi’ limma's arrayWeights function. Analytes with an estimated absolute fold difference of more than 20% at a false discovery rate of less than 10% were deemed significant. To visualize adjusted abundances in the heatmap, the batch, sex and SV covariates were regressed out with limma's removeBatchEffect function. LC/MS results from methoxy-positive and -negative microglia were analyzed in a similar way, but as no significant surrogate variables were identified, median scaling was applied to the log_2_ transformed ratios of each sample instead and the following linear model was fit using limma: log_2_(abundance) ~ genotype + batch + sex.

### Data availability

RNA-seq datasets have been deposited online in the Gene Expression Omnibus repository as SuperSeries GSE158156, which is composed of the SubSeries GSE158152 and GSE158153

## Results

### Generation of the *App*^SAA^ KI mouse model and characterization of APP metabolism

**Fig. 1:**
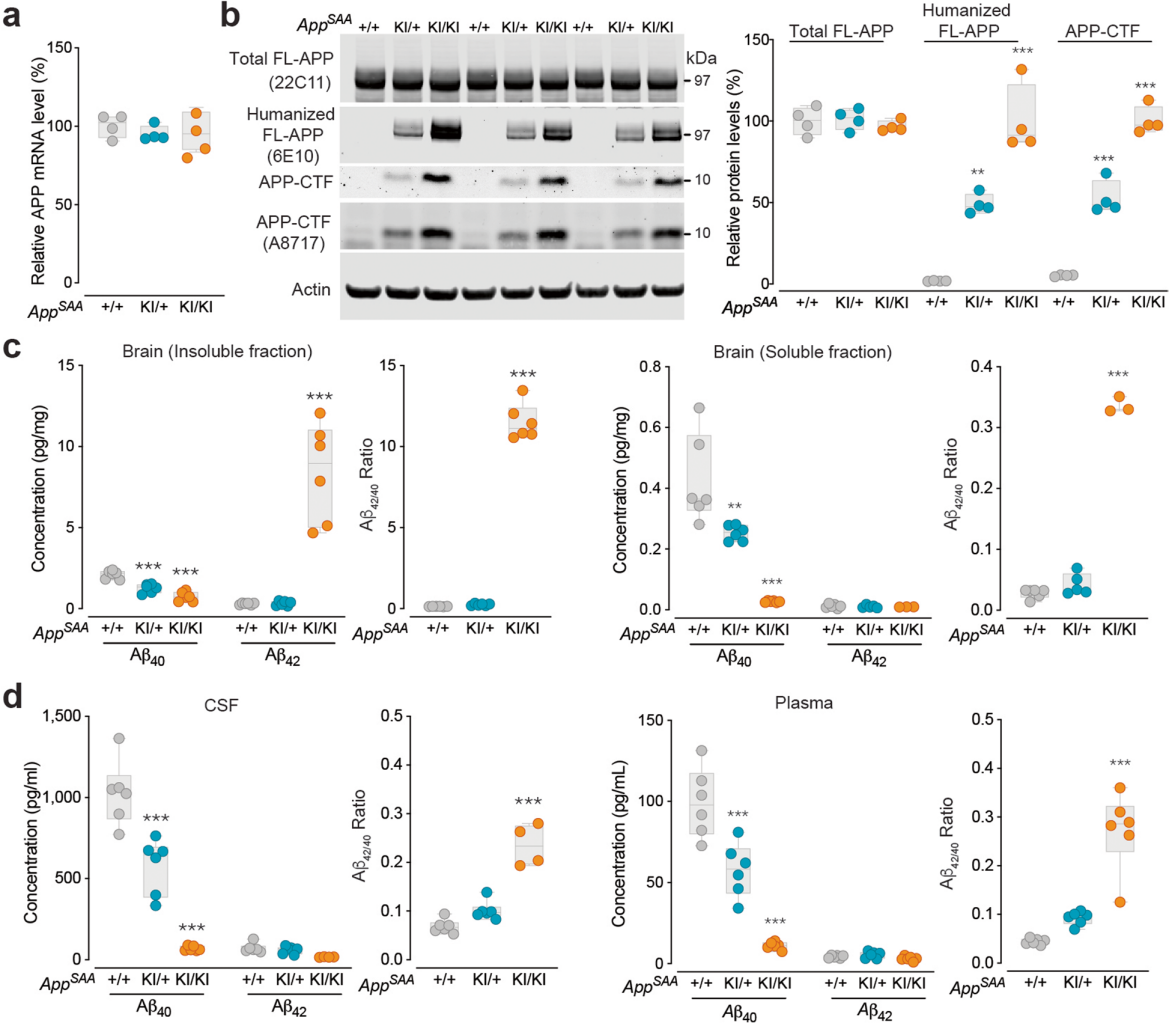
Alterations of APP cleavage and Aβ levels in *App*^SAA^ knock-in mice. **a** RT-qPCR analysis of brain RNA shows normal *App* mRNA level in the *App*^SAA^ homozygous mice (KI/KI), heterozygous mice (KI/+) and wild-type mice (+/+) at 2-month-old. **b** Western blotting analysis of brain lysates at 2-month-old shows normal expression of full-length APP proteins in all three genotypes and KI gene dosage-dependent increase of human full-length APP proteins and APP-CTF in KI/KI and KI/+ mice. β-Actin was used as internal loading control. **c** Measurement of Aβ40 and Aβ42 in brain insoluble and soluble fractions extracted from animals at 4 months of age. Level of insoluble Aβ42 is increased in KI/KI homogenates and level of insoluble Aβ40 is reduced in KI/KI and KI/+ homogenates relative to wild-type control. The insoluble Aβ42/Aβ40 ratio is significantly enhanced in KI/KI brains. Level of soluble Aβ42 is unchanged in KI/KI homogenates and level of soluble Aβ40 is reduced in KI/KI and KI/+ homogenates relative to wild-type control. The soluble Aβ42/Aβ40 ratio is significantly increased in KI/KI brains. **d** Measurement of Aβ40 and Aβ42 in CSF and plasma collected from mice at 4 months of age. Level of Aβ42 is unchanged in KI/KI CSF while level of Aβ40 is reduced in KI/KI and KI/+ CSF relative to wild-type control. The CSF Aβ42/Aβ40 ratio is significantly increased in KI/KI CSF. Level of Aβ42 is unchanged in KI/KI plasma while level of Aβ40 is reduced in KI/KI and KI/+ plasma relative to wild-type control. The plasma Aβ42/Aβ40 ratio is significantly increased in KI/KI. Graphs are box and whisker plots and P values: one-way ANOVA with Dunnett’s multiple comparison test, each group compared to the *App*^SAA^ +/+ control group; **P < 0.01, and ***P < 0.001. Sample size: 3-6 per genotype.

We used a knock-in (KI) strategy to humanize the Aβ sequence of the murine *App* gene and introduced three fAD mutations - Swedish (KM670/671NL) [54], Arctic (E693G) [55] and Austrian (T714I) [56] - using homologous recombination (Supplementary Fig. 1a). To determine whether introduction of the fAD and humanization mutations disrupts *App* expression, we examined *App* mRNA levels in *App*^SAA^ KI/KI (homozygous), *App*^SAA^ KI/+ (heterozygous) and *App*^SAA^ +/+ (wild type) mice at 2 months of age. RT-qPCR analysis showed normal levels of *App* mRNA in KI/KI and KI/+ brains (Fig. 1a). Western blotting analysis with an antibody recognizing the N-terminus of mouse and human APP (22C11) revealed similar full-length APP protein levels across the three genotypes (Fig. 1b). Immunoblotting with an antibody recognizing amino acids 1-16 of human Aβ (6E10) detected the humanized Aβ sequence in the full-length APP and C-terminal APP fragments (APP-CTF) in brains of KI/+ animals, and we observed elevated levels in the KI/KI background (Fig. 1b), as expected based on enhanced cleavage due to the Swedish mutation [57, 58]. The elevation of APP-CTF was further validated by antibody A8717 which specifically recognizes the C-terminus of APP (Fig. 1b). These results demonstrate that *App*^SAA^ does not affect full-length APP expression but alters its cleavage products.

**Fig. 2:**
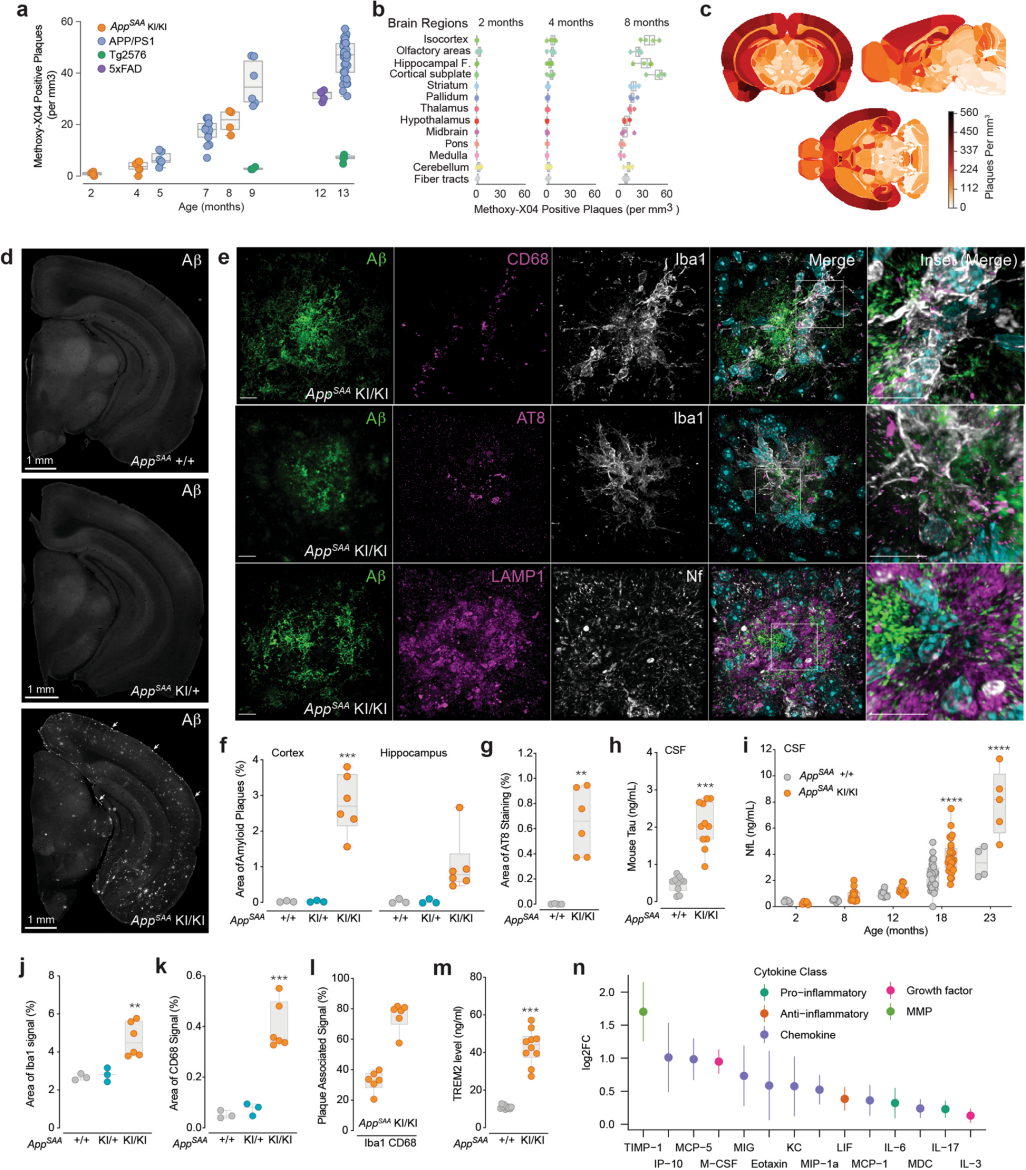
Amyloid plaque pathology, biomarkers of neurodegeneration and neuroinflammation. **a** Amyloid plaques were measured by segmentation and registration to the CCFv3 atlas post-methoxy-X04 labeling. *N* = 4-30 mice/ group. **b** Methoxy-X04 positive plaque density across brain regions in the *App*^SAA^ KI/KI. Hippocampal F. = formation. N = 4-6 mice/ group. **c** 3D heatmaps show brain distribution of methoxy-X04 positive plaques in *App*^SAA^ KI/KI at 8-month-old. Except panel **i**, all panels from **d-n** show data from 8-month-old mice. **d** Representative images of brain sections show Aβ plaque immunoreactivity in *App*^SAA^ KI/KI but not in KI/+ mice. Leptomeningeal cerebral amyloid angiopathy is indicated by arrows. **e** Confocal images from *App*^SAA^ KI/KI stained for plaques, microglia markers (Iba1, CD68), dystrophic neurites (AT8), neurofilament (Nf) and lysosomal marker (LAMP1). Far right column provides a magnified view of the inset in merged images. Scale bars = 10 μm. **f-g** Quantification of brain areas covered by Aβ plaques (**f**) and AT8 area (**g**). N = 3-6 mice/ genotype. **h** Total tau levels in CSF. *N* = 6-11 mice/ genotype. **i** Higher level of CSF Nf-L in aged *App*^SAA^ KI/KI relative to +/+ mice. *N* = 4-33 mice/ group. Age: F (4, 111) = 61.74, *P* < 0.0001. Genotype: F (1, 111) = 33.46, *P* < 0.0001. **j-l** Quantification of Iba1 (**j**), CD68 (**k**), percentage of the Iba1 and CD68 signals overlapping with plaques (**l**). *N* = 3-6 mice/ genotype. **m** TREM2 levels measured in brain homogenates. *N* = 10 mice/ genotype. **n** Differential abundance (log2) of cytokines measured in brain lysates. *N* = 10 mice/ genotype. Bars represent 95% confidence intervals of fold change. Graphs from **f-m** are box and whisker plots. P values: (**f, j, k**) one-way ANOVA with Dunnett’s multiple comparison test, (**g, h, m**) t-test. Each genotype compared to *App*^SAA^ +/+; **P < 0.01, ***P < 0.001 and ****P < 0.0001

We then used an immunoassay with the 4G8 antibody to measure Aβ_40_ and Aβ_42_ in *App*^SAA^ mice. The Aβ_42/40_ ratio was increased in soluble and insoluble brain fractions, CSF, and plasma from *App*^SAA^ KI/KI at 2 and 4 months of age (Fig. 1c, d and Supplementary Fig. 1b-e). At 2 months of age, before amyloid plaques were detected (Fig. 2a), we observed a reduction of Aβ_40_ levels in brain fractions, CSF and plasma from *App*^SAA^ KI/KI, leading to an increased Aβ_42/40_ ratio (Supplementary Fig. 1b-e). At 4 months of age, an increase of Aβ_42_ levels in insoluble brain fraction further increased the Aβ_42/40_ ratio in the *App*^SAA^ KI/KI (Fig. 1c). The reduction of Aβ_40_ levels in brain and biofluids in young *App*^SAA^ KI/KI mice could be induced by the Austrian fAD mutation, which has been reported to impact APP cleavage by γ-secretase [56]. Alternatively, it is possible that the 4G8 antibody used in this assay has reduced binding to humanized Aβ (in *App*^SAA^ KI/KI) as compared to murine Aβ (in *App*^SAA^ +/+), leading to an apparent decrease in Aβ_40_. However, since 4G8 antibody similarly binds Aβ_40_ and Aβ_42_, the calculated Aβ_42/40_ ratio for both *App*^SAA^ +/+ controls and *App*^SAA^ KI/KI mice are independent of the affinity of 4G8 for humanized versus murine Aβ.

### Progression of amyloid-plaque pathology and associated neurodegeneration

Methoxy-X04 is a blood-brain permeable fluorescent dye, derived from congo red, that is widely used to stain amyloid plaques containing fibrillar forms of Aβ [59, 60]. To analyze the spatial distribution of Aβ deposition at the whole brain level, we injected methoxy-X04 intraperitoneally in KI/KI, KI/+ and +/+ mice from 2 to 8 months of age and euthanized animals 24 hours post injection for two-photon serial imaging [31]. Amyloid deposition was detected in *App*^SAA^ KI/KI from 4 months of age and the total brain density of Aβ plaques increased in an age-dependent manner at 4 and 8 months of age in this genotype (Fig. 2a). In contrast, no methoxy-X04 positive plaques were observed in *App*^SAA^ KI/+ from 4 to 8 months. Aβ deposition in *App*^SAA^ KI/KI was comparable to that reported for commonly used transgenic models, such as the APP/PS1 and the 5xFAD lines (Fig. 2a). At 8 months of age, Aβ plaques were detected in multiple brain regions, but the highest density of pathology was found in cortical and hippocampal regions (Fig. 2b, c). Comparing the temporal and spatial plaque deposition patterns in *App*^SAA^ KI/KI with progression of amyloid pathology in human AD [31, 61] revealed remarkable similarities in plaque distribution (Supplementary Fig. 2). Immunostaining with an anti-amyloid antibody confirmed the age-dependent amyloid pathology in the *App*^SAA^ KI/KI brains (Fig. 2d, f; Supplementary Fig. 3a, b) and identified plaques in the *App*^SAA^ KI/+ brains at 16 months of age (Supplementary Fig. 5a). No sex effect was detected in amyloid burden in the cortex (2-way ANOVA: p=0.49, 4 males and 2 females) or the hippocampus (2-way ANOVA: p=0.31, 4 males and 2 females) in 8 month-old *App*^SAA^ KI/KI mice (Fig. 2f), but since sample sizes were relatively small in this analysis, a potential sex effect cannot be firmly excluded. Moreover, anti-Aβ and methoxy-X04 co-stainings revealed a lower percentage of methoxy-X04 positive plaques in the *App*^SAA^ KI/KI brains compared to that in 5xFAD brains at 6-8 months of age (Supplementary Fig. 4a, b), indicating that the plaques in *App*^SAA^ KI/KI brains are less dense than in the 5xFAD brains. We also identified vascular Aβ deposition in *App*^SAA^ KI/KI at 8 and 16 months of age, particularly in leptomeningeal (pial) vessels at the brain surface, demonstrating the presence of cerebral amyloid angiopathy (CAA) in this model (Fig. 2d, Supplementary Fig. 5b-e).

Swollen, dystrophic neural processes surrounding amyloid plaques are a common pathological feature of AD [62]. To determine if amyloid plaques were associated with this type of neurodegenerative processes in *App*^SAA^ KI/KI, we analyzed three markers of dystrophic neurites by immunohistochemistry at 8 months of age: phosphorylated tau (pTau), neurofilament (axonal marker) and LAMP1 (lysosomal marker). We observed numerous AT8-positive pTau puncta (Fig. 2e, g), abnormal shape of neurites (swollen, expanded, dystrophic) and accumulation of LAMP1 (Fig. 2e) around plaques, which are all prominent features of dystrophic neurites.

To further explore if amyloid pathology caused elevation of biomarkers of neurodegeneration in *App*^SAA^ KI mice, we measured total tau and neurofilament light chain (Nf-L) in CSF. We found that total tau levels were elevated in *App*^SAA^ KI/KI relative to the *App*^SAA^ +/+ controls at 8 months of age (Fig. 2h). We also observed age-dependent increase of CSF Nf-L in both *App*^SAA^ KI/KI and *App*^SAA^ +/+ from 2 to 23 months of age (Fig. 2i). Levels of Nf-L were significantly higher in *App*^SAA^ KI/KI mice at 18 and 23 months of age when compared to *App*^SAA^ +/+ (Fig. 2i). No sex difference was detected in Nf-L levels across ages (ANOVA, sex variable, p=0.49). Altogether, our data demonstrate that the *App*^SAA^ KI mouse model develops progressive deposition of neuritic plaques that is associated with an elevation of neurodegeneration biomarkers.

To investigate microglial responses, we stained brain sections for Iba1 and CD68 proteins in the cortex and the hippocampus from *App*^SAA^ KI/KI at 8 months of age. We found that microglia density was increased in the vicinity of amyloid plaques with an enrichment in CD68-positive microglia (Fig. 2e, j-l and Supplementary Fig. 6a-d), indicating clustering of responsive microglia around plaques. Additionally, levels of TREM2 and various cytokines were elevated in brain homogenates from *App*^SAA^ KI/KI at 8 months of age (Fig. 2m, n), showing profound immune responses.

One of the hallmarks of AD and related animal models is reactive astrogliosis, a process characterized by complex molecular, morphological, and functional changes in astrocytes [63]. To assess astrocytic changes in 18-month-old *App*^SAA^ KI/KI mice, we first analyzed the expression of glial fibrillary acidic protein (GFAP), a commonly used marker of astrocytic responses in disease. We found robust and widespread increases in GFAP immunoreactivity in *App*^SAA^ KI/KI mice as compared to age matched *App*^SAA^ +/+ controls, especially in the neocortex and hippocampus (Supplementary Fig. 7a, d). We also detected strong polarization of GFAP-immunoreactive astrocytes around Aβ plaques in *App*^SAA^ KI/KI mice. Next, we assessed the expression of GLT-1 (also known as Slc2a1, or excitatory amino acid transporter 2 (EAAT2)), a major glutamate transporter mainly expressed by astrocytes and essential for synaptic glutamate homeostasis [64, 65]. Decreased GLT-1 levels have been previously reported in postmortem AD brain tissue [66–68], suggesting that AD pathology involves dysregulation in excitatory transmission. In agreement with previous reports, we found decreased levels of GLT-1 immunoreactivity in the hippocampus of *App*^SAA^ KI/KI mice (Supplementary Fig. 7b, e). Lastly, we assessed the expression of C3, a complement factor implicated in AD pathology and upregulated in astrocytes in response to microglial release of proinflammatory factors [69, 70]. Similar to GFAP, astrocytic C3 levels were increased in the hippocampus, neocortex, and other brain regions of *App*^SAA^ KI/KI mice, and these increases seemed highest near Aβ plaques (Supplementary Fig. 7c, f). Together, these results suggest that *App*^SAA^ KI/KI mice have profound alterations in astrocytes that may contribute to disease progression in this model.

### Multi-dimensional phenotyping of the microglial response

**Fig. 3:**
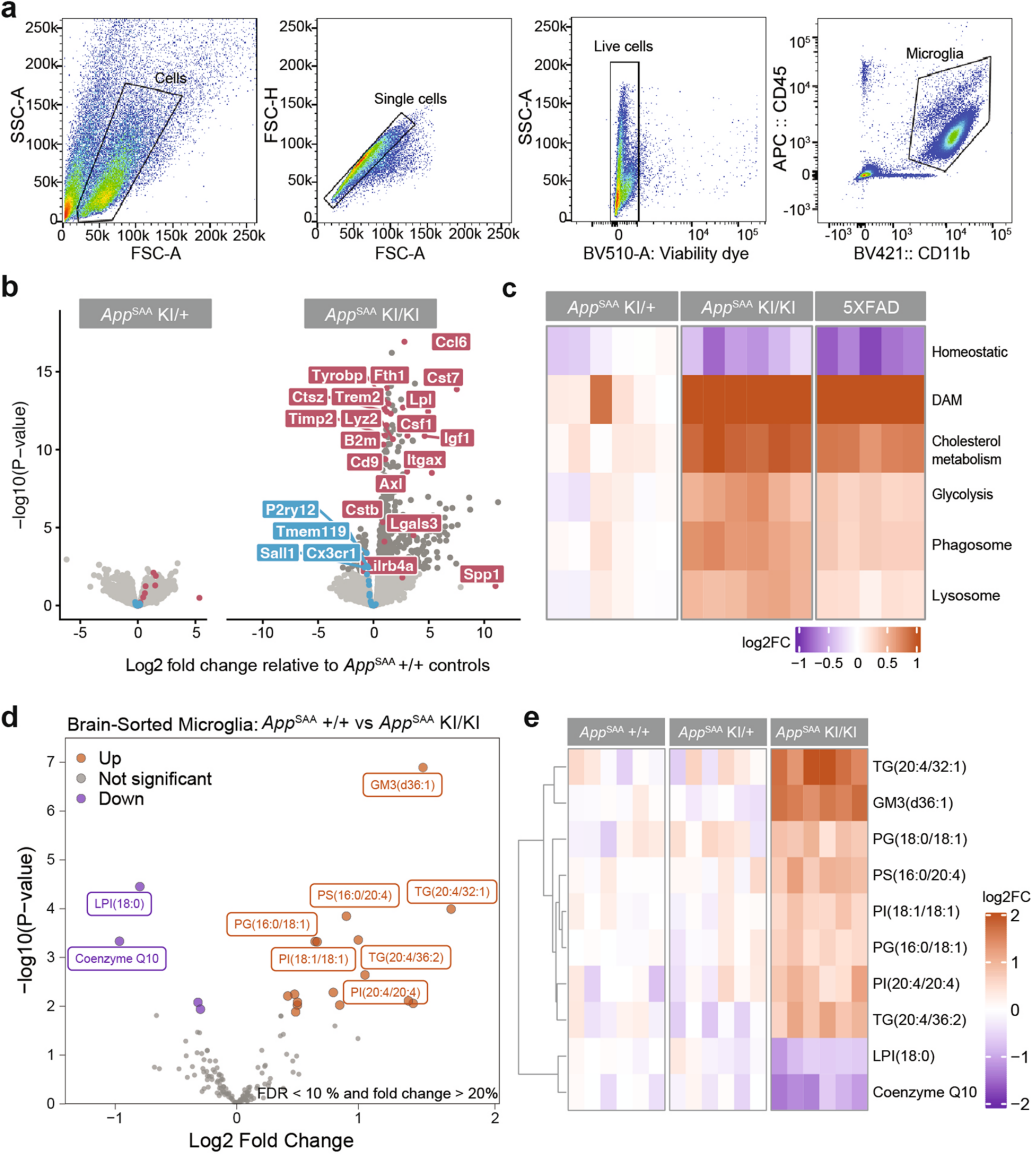
Multi-omics analysis of microglia from *App*^SAA^ KI/KI and control mice at 8 months of age. **a** FACS gating strategy for isolating microglia from whole brains of *App*^SAA^ homozygous mice (KI/KI), heterozygous mice (KI/+) and wild-type mice (+/+) at 8 months of age for transcriptomics, lipidomics, and metabolomics analysis. **b** Volcano plots showing log2 fold change of gene expression between *App*^SAA^ heterozygous (left) and homozygous (right) knock-in compared to WT; dark grey, fuchsia, and blue indicate DEGs (FDR <= 10%; see methods), DAM, and homeostatic genes, respectively. *N* = 6 mice per genotype. **c** Single sample activity scores for curated gene sets in *App*^SAA^ heterozygous, *App*^SAA^ homozygous, and 5xFAD. Intensities indicate log2 fold change of aggregated gene set score (row) per mouse (column) with respect to mean gene set score from matched WT mice. *N* = 5-6 mice per genotype. **d** Volcano plot showing log2 fold change of lipids between *App*^SAA^ homozygous mice compared to WT. FDR <= 10%; absolute fold change > 20%. *N* = 6 mice per genotype. **e** Heatmap of top 10 lipids significantly altered by genotype in isolated microglia; columns represent individual mice. Intensities indicate log2 fold change of lipids with respect to mean abundance in WT mice. *N* = 6 mice per genotype

The presence of microglia clusters around amyloid plaques and the elevation of cytokines suggested that microglia transitioned to a responsive state. To further characterize this response, we isolated CD11b^+^ microglia from *App*^SAA^ KI/KI, KI/+ and +/+ mice at 8 months of age by FACS (Fig. 3a) and analyzed their mRNA, metabolite, and lipid profiles as previously described in non-amyloid models [22].

We first confirmed the enrichment and purity of isolated microglia using RNA-seq data to compare the expression levels of previously reported marker genes from different cell types in the mouse brain [71] (Supplementary Fig. 8a). At 8 months of age, the microglial transcriptome from *App*^SAA^ KI/+ showed little difference when compared to *App*^SAA^ +/+ controls (Fig. 3b). Microglia from *App*^SAA^ KI/KI mice, however, contained more than six hundred differentially expressed genes (DEGs) *vs. App*^SAA^ +/+ controls (absolute log2 fold change [FC] > 1.2, false discovery rate [FDR] < 10%), with an upregulation of disease-associated microglia (DAM) genes (Fig. 3b). Increased activity of other gene sets related to microglial state and function, such as cholesterol metabolism, glycolysis, phagocytic and lysosomal function resembled those observed in microglia from the 5xFAD transgenic or demyelination models [14, 22, 43] (Fig. 3c), demonstrating that some aspects of the microglia signature are conserved across diverse APP mouse models, including the *App*^SAA^ KI mouse model.

Targeted liquid chromatography and mass spectrometry (LCMS) analysis of 176 lipids in FACS-isolated microglia identified 4 significantly decreased and 16 significantly increased analytes between *App*^SAA^ KI/KI and +/+ mice (>= 20% change with FDR < 10%) (Supplementary Tables 5 and 6). Ganglioside GM3 (d36:1), various species of triglyceride (TG) (including species of arachidonate-containing TG) as well as a variety of phospholipid species (*i.e.*, PG, PS and PI) were more abundant in *App*^SAA^ KI/KI microglia (Fig. 3d-e). Conversely, lysolipid lysophosphatidylinositol LPI 18:1 and coenzyme Q10 were less abundant in *App*^SAA^ KI/KI microglia (Fig. 3d-e). Together, these observations indicate broad changes in microglia lipid metabolism in the *App*^SAA^ KI mouse model, reminiscent of findings in human AD brains [72] and other AD mouse models [73, 74].

### Methoxy-X04-positive microglia exhibit an exacerbated gene expression and metabolic response

**Fig. 4:**
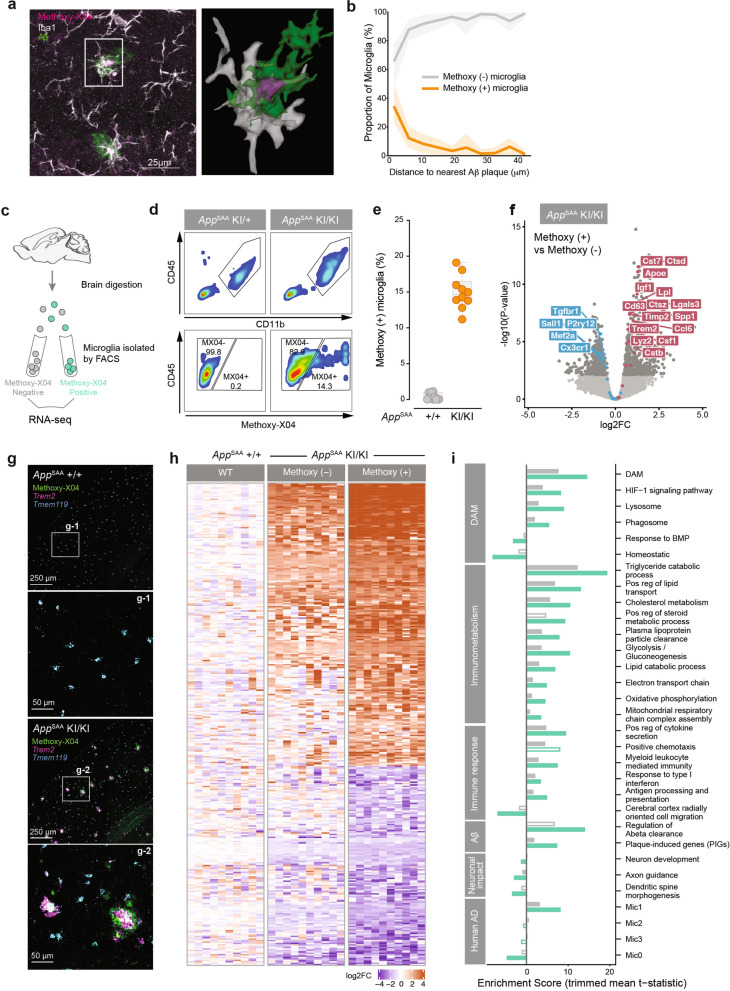
Methoxy-X04 (+) microglia from *App*^SAA^ KI/KI mice exhibit exacerbated gene expression and metabolic profiles. **a** (Left) Confocal images of cortex from 8-month-old *App*^SAA^ homozygous mice (KI/KI) stained for amyloid plaques and microglia markers (Iba1) post methoxy-X04 injection. Scale bars = 25 μm. (Right) a magnified view of the inset. **b** Quantification of methoxy-X04 (+) microglia proximal to plaques n=6. **c-d** Schematic of FACS experiment and gating strategy used to isolate pure populations of microglia that are negative (-) or positive (+) for methoxy-X04. **e** Fraction of methoxy-X04 (+) microglia purified from WT and *App*^SAA^ KI/KI mouse brains at 8 months of age. *N* = 10. **f** Volcano plot showing log2 fold change of gene expression between methoxy-X04 (+) and methoxy-X04 (-) samples. Genes expressed higher in methoxy (+) microglia have log2 fold changes > 0; colors same as in Fig. 3b. **g** Images of brain sections showing methoxy-X04 labeling and detection of *Trem2* and *Tmem119* transcripts by *in situ* hybridization. **h** Gene expression profiles from a representative subset of differentially expressed genes in methoxy-X04 (-) and (+) *App*^SAA^ KI/KI microglia when compared to WT. Intensities correspond to the log2 fold- change for each gene (row) per sample (column) as compared to the mean expression of the gene in the methoxy-X04 (-) WT group. **i** Gene set enrichment analysis from methoxy-X04 (-) (grey) or methoxy-X04 (+) (light green) *App*^SAA^ KI/KI vs methoxy-X04 (-) WT mice. Enrichment scores are calculated as the mean t-statistic of genes in the leading edge of the gene set. Filled bars indicate gene set enrichment results with FDR <= 10%

Next, we asked whether proximity to amyloid plaques affected microglia gene expression, lipid, and metabolite profiles. We hypothesized that the microglia in proximity to amyloid plaques may have more phagocytic activity and show higher intracellular Aβ levels than the microglia distal to plaques. We labelled fibrillar Aβ in *App*^SAA^ KI/KI by peripheral injection of methoxy-X04 and quantified the proximity of methoxy-X04-positive (+) microglia to amyloid plaques via super-resolution microscopy (Fig. 4a). We confirmed that most methoxy-X04 (+) microglia were found around amyloid plaques (Fig. 4b), consistent with the notion that those microglia phagocytosed Aβ.

To further characterize the relationship between microglial phagocytic activity, gene, and metabolite profiles, we sorted methoxy-X04 (-) and (+) microglia by FACS prior to conducting RNA-seq, lipidomic, and metabolomic analyses (Fig. 4c–d). We found that about 15% of microglia are methoxy-X04 (+) (Fig. 4e) in *App*^SAA^ KI/KI at 8 months of age. This figure is comparable to what was reported in the 5xFAD model at 4 months of age [75]. Direct comparison of methoxy-X04 (+) *vs.* (-) microglia from *App*^SAA^ KI/KI identified more than 800 DEGs, with methoxy-X04 (+) microglia showing increased DAM activation (Fig. 4f), as well as increased expression of *Gusb, Prdx6, Ctsa, Arpc1b, Grn* among other genes recently reported in the plaque-induced gene (PIG) signature [8] (Supplementary Fig. 8b). Higher levels of *Trem2* transcripts were detected in methoxy-X04 positive amyloid plaques by *in situ hybridization* (Fig. 4g) in *App*^SAA^ homozygous mice at 8 months of age, consistent with an enrichment of responsive microglia clustering around amyloid plaques (Fig. 2k-l). Methoxy-X04 (-) microglia from *App*^SAA^ mice also showed significant changes compared to methoxy-X04 (-) microglia from WT control mice, but the effects were not as strong as the ones measured in *App*^SAA^ methoxy-X04 (+) microglia (Fig. 4h; Supplementary Fig. 8b).

Gene set enrichment analysis (GSEA) revealed approximately twice as many significant pathways (FDR <= 10%) in the methoxy-X04 (+) relative to the methoxy-X04 (-) microglia when these populations were compared against WT. Most notably, methoxy-X04 (+) cells exhibited higher enrichment for genes regulating innate immune function as well as metabolic functions that appear critical for supporting lipid clearance and metabolism (Fig 4i). As anticipated, these cells also showed an enrichment in genes associated with regulation of Aβ clearance and amyloid plaques (PIGs) (Fig 4i). Finally, the upregulation of the signature genes (*Spp1*, *Tmem163*, *Apoe*, *Ctsb*, *Hif1a*, among others) from the Mic1 microglia sub-cluster in human AD [19] were enriched in the phagocytic microglia from the *App*^SAA^ homozygous mice (*p-*value < 1e-4, hypergeometric test; Fig 4i), suggesting that this mouse model successfully recapitulates a subset of the microglial response found in human disease.

**Fig. 5:**
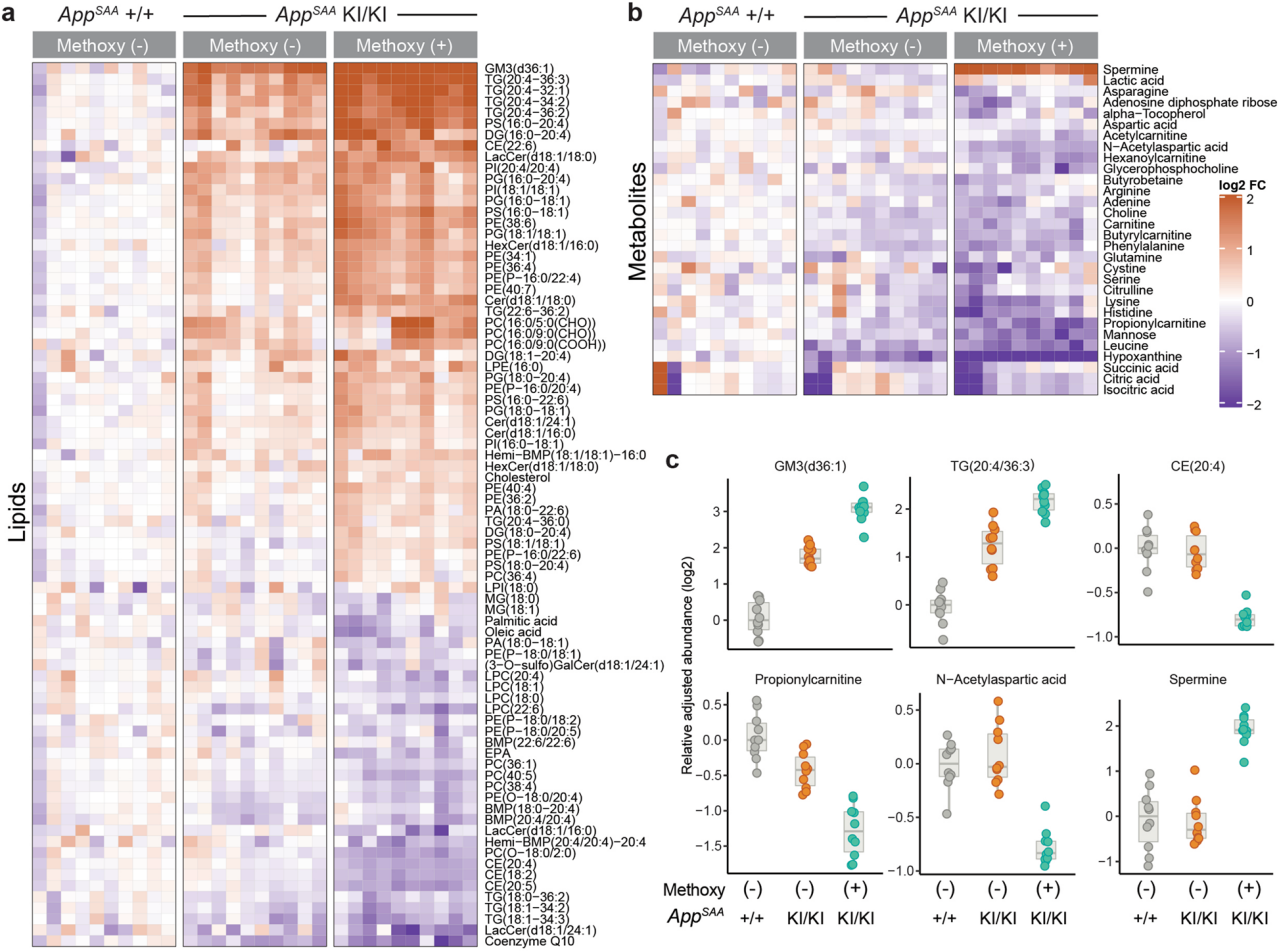
Methoxy-X04 (+) microglia isolated from *App*^SAA^ knock-in mice show alterations of lipids and metabolites levels. **a-b** Heatmap of (**a**) lipids and (**b**) metabolites significantly altered by genotype and/or presence of methoxy-X04 dye in microglia isolated from *App*^SAA^ KI/KI and *App*^SAA^ +/+ mouse brain. n=10, columns represent individual mice. **c** Representative plots showing genotype and methoxy-X04 effects on key lipids and metabolites from FACS-isolated microglia. *n*=10. Statistical analysis was performed by fitting linear models using limma; FDR was calculated according to Benjamini’s and Hochberg’s method. All panels from **a-c** are showing data from 8-month-old mice

Mirroring our transcriptomic analyses, lipid alterations were exacerbated in *App*^SAA^ methoxy-X04 (+) microglia relative to methoxy-X04 (-) and WT microglia, as shown for instance for GM3(d36:1) and TG 20:4/36:3 species (Fig. 5a, Supplementary Tables 7 and 8). In total, 48 of the 172 assayed lipids showed higher and 32 displayed lower abundance in *App*^SAA^ methoxy-X04 (+) compared to WT microglia (>= 20% change with FDR < 10%). Some lipid changes appeared to be specific to methoxy-X04 (+) microglia, including an increase in cholesteryl ester CE 22:6 and a decrease in other CE species, such as CE 20:4 (Fig. 5a). Finally, analyses of 101 polar metabolites in sorted microglia revealed 2 analytes with increased and 28 with decreased abundance in *App*^SAA^ methoxy-X04 (+) compared to WT microglia (>= 20% change with FDR < 10%; Supplementary Tables 7 and 8). For example, the polyamine spermine accumulated specifically in methoxy-X04 (+) microglia (Fig. 5b-c). Therefore, our data lends further support to the notion that phagocytic microglia undergo profound cellular alterations, including changes in lysosomal activity, lipid dyshomeostasis, and other metabolic changes.

### *App*^SAA^ mice show enhanced TSPO and FDG-PET signals in brain

**Fig. 6:**
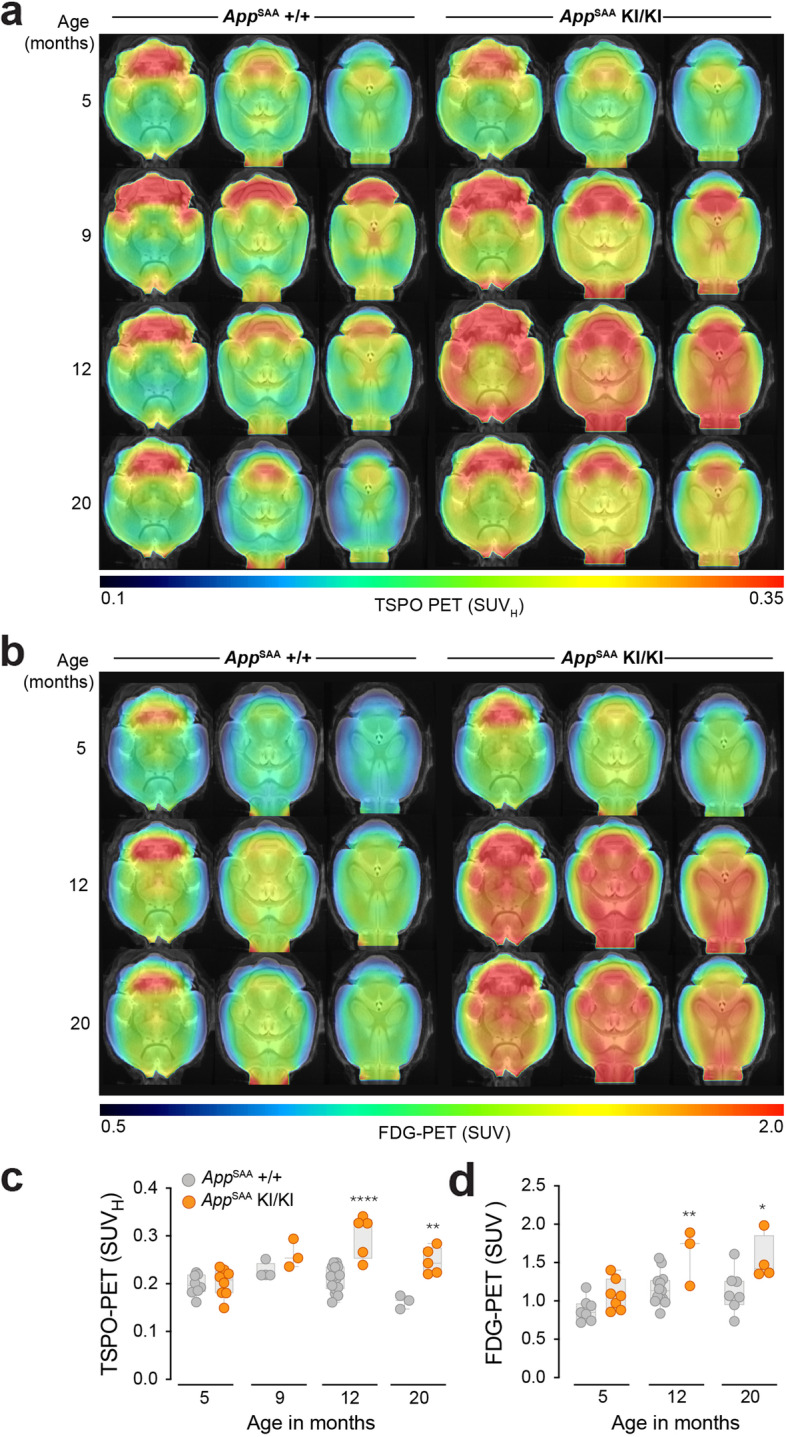
Elevated TSPO- and FDG-PET cortical signals in *App*^SAA^ mice. **a-b** Axial slices upon an MRI template show TSPO-PET and FDG-PET uptake patterns of WT and *App*^SAA^ mice at different age groups. **c** *App*^SAA^ KI/KI mice had a significantly higher TSPO-PET signal at 12 months and 20 months of age when compared to *App*^SAA^ +/+ control mice. **d** *App*^SAA^ KI/KI mice had a significantly higher glucose uptake at 12 and 20 months of age when compared to *App*^SAA^ +/+ control mice. P values: t-test; *P < 0.05, **P < 0.01, and ****P < 0.0001.

Based on the amyloid-related response of microglia in the *App*^SAA^ KI model, we sought to determine if mice show elevated levels of the 18KDa translocator protein TSPO, an imaging marker that is associated with microglia biology [20, 76, 77] and elevated in AD patients [78, 79]. We compared the TSPO positron emission tomography (PET) signal of *App*^SAA^ +/+ controls and *App*^SAA^ KI/KI at different ages. *App*^SAA^ KI/KI mice had a significantly higher TSPO-PET signal in the cortex at 12 and 20 months of age when compared to *App*^SAA^ +/+ mice (Fig. 6a, c). To evaluate if this increase in TSPO expression was associated with changes in brain metabolism, we measured glucose uptake by 2-deoxy-2- [^18^F]fluoro-d-glucose (FDG)-PET. The cortical signal of FDG-PET was also elevated in *App*^SAA^ KI/KI compared to *App*^SAA^ +/+ across the studied lifespan (Fig. 6b). *App*^SAA^ KI/KI had a significantly higher glucose uptake at 12 and 20 months of age when compared to control mice (Fig. 6d). TSPO-PET and FDG-PET quantification were strongly associated in *App*^SAA^ KI/KI (R=0.709, p=0.007) but not in *App*^SAA^ +/+ (R=0.171, p=0.577), suggesting that the elevation of glucose metabolism could be partially driven by responsive microglia in this model, as recently demonstrated in a distinct mouse model [20].

### Characterization of behavioral phenotypes in *App*^SAA^ KI model

**Fig. 7:**
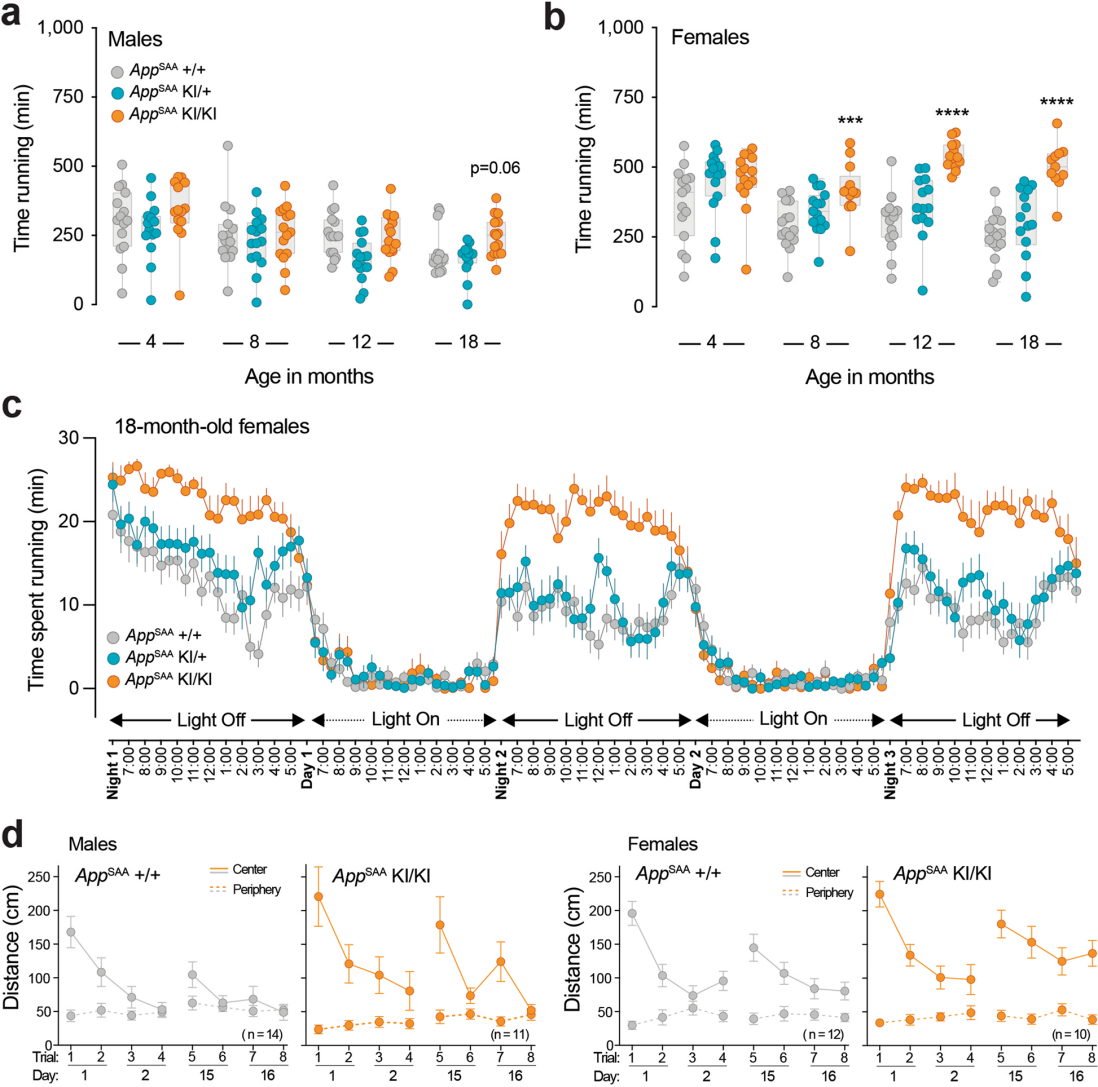
Hyperactivity phenotype in *App*^SAA^ KI/KI mice. **a-c** Spontaneous activity was monitored in home-cage using running wheels. The average time running is represented for males (**a**) and females (**b**) at 4, 8, 12 and 18 months of age for the three genotypes (*App*^SAA^ +/+, KI/+ and KI/KI). Graphs are box and whisker plots. P values: one-way ANOVA with Dunnett’s multiple comparison test. *App*^SAA^ KI/KI compared to the *App*^SAA^ +/+ control group; ***P < 0.001 and ****P < 0.0001. **c** Illustration of time spent running averaged per genotype in female mice at 18 months of age. Data are represented as mean ± SEM. **d** 16–17-month-old male and female *App*^SAA^ mice (KI/KI) and wildtype littermate controls (+/+) were tested in the open field for 5 min twice a day for two days (Day 1–2; Trials 1–4) and retested two weeks later (Day 15–16; Trials 5–8). Top: Distance travelled (cm) in the center and periphery of a circular arena. Bottom: P values for the repeated three-way ANOVA with genotype (KI/KI vs +/+), sex (male vs female), and location (center vs periphery) as fixed factors and their interactions. Bars represent mean ± SEM. Number of mice indicated in parentheses

APP transgenic mouse models often display behavioral alterations, including locomotor hyperactivity, habituation deficits to a novel environment, emotional alterations and learning and memory deficit s[80, 81]. The emergence of behavioral abnormalities in *App* KI mouse models indicates that accumulation of Aβ without APP overexpression is sufficient to trigger age-dependent brain dysfunction [7, 82, 83]. To determine whether the *App*^SAA^ KI mouse model developed similar behavior alterations, we first evaluated a cohort of mice from 4 to 18 months of age using the frailty scoring, activity monitoring in their home cage and an open field. *App*^SAA^ KI/KI and KI/+ did not show deficits in frailty index at any of the tested ages (Supplementary Table 9), indicating that this model does not develop major general health issues. *App*^SAA^ KI/KI mice displayed a robust hyperactivity phenotype at 18 months of age in the open field (Supplementary Table 9). Longitudinal monitoring of running wheel activity in their home cages revealed an age-dependent increase in spontaneous activity (Fig. 7a-c). Females showed a more pronounced phenotype compared to males, with a significant hyperactivity phenotype detected at 8 months of age (Fig. 7a-c). To determine whether *App*^SAA^ KI/KI show any deficit in habituation to a novel environment, we tested an independent cohort of mice at 16–17-month-old in a repeated version of the open field (Fig. 7d). Mice were tested twice a day for two days (trials 1–4) and retested two weeks later (trials 5–8) to assess locomotor activity and short- and long-term habituation [84]. Compared to sex-matched wildtype littermate mice, *App*^SAA^ KI/KI had increased locomotor activity (hyperactivity), which became more prominent as wildtype mice habituated to the open field over the repeated testing (habituation deficits), and *App*^SAA^ KI/KI showed stronger center preference (disinhibition). Overall, there were prominent effects in genotype (*p* = 0.004), sex (*p* = 0.008), and location (*p* <0.001), indicating that *App*^SAA^ KI/KI displayed altered behavior; males and females regardless of genotype behaved differently; and there was a distinct preference for location (center). Genotype interacted with location (*p* < 0.001), but not with sex (*p* = 0.439), indicating that *App*^SAA^ KI/KI behaved differently in the center (higher activity) than in the periphery (decreased activity) relative to wildtype littermate controls, and that male and female *App*^SAA^ KI/KI displayed similar behavioral alterations relatively to sex-matched wildtype mice. Notably, male *App*^SAA^ KI/KI displayed prominent hyperactivity during the first (Trials 5 and 7) but not the second (Trials 6 and 8) trials of the retest days, whereas female *App*^SAA^ mice displayed a more consistent hyperactivity throughout all trials relative to sex-matched wildtype controls. These results indicate that aged male and female *App*^SAA^ KI/KI develop robust hyperactivity, habituation deficits, and disinhibition alterations.

## Discussion

This study demonstrates that a mouse model expressing physiologically relevant levels of APP bearing familial AD mutations recapitulates several key aspects of AD biology. The *App*^SAA^ KI mouse model shows progressive accumulation of amyloid pathology, increased levels of biomarkers of neurodegeneration, and robust astroglial and microglial responses. Deep phenotyping of microglia by multi-omics approach demonstrates that phagocytic microglia in the vicinity of amyloid plaques display profound gene expression and metabolic dysregulation in this model. Given the large contribution of microglial genes to AD risk, and the many microglial-directed therapies currently in clinical development [10] this model represents an opportunity to gain novel insight into the role of microglial biology in the context of amyloid pathology and offers a translationally relevant model system for evaluating the impact of microglial-directed therapies in a disease context.

As anticipated based on the diffuse amyloid deposits seen in AD patients carrying the Austrian or the Arctic mutation [85], a large proportion of the amyloid plaques in the *App*^SAA^ mice were also diffuse with or without a dense core structure. The fibrillar form of Aβ combined with a diffuse halo of amyloid was associated with key features of neuritic plaques [86, 87] in the *App*^SAA^ mice, including dystrophic neurites, accumulation of phosphorylated tau and enlarged LAMP1-positive lysosomes. In response to the progressive accumulation of amyloid plaques, the *App*^SAA^ mice developed a robust immune response that was characterized by elevated levels of multiple neuroinflammatory cytokines and TREM2 in brain lysates, an increased density of responsive microglia at the vicinity of amyloid plaques, profound expression changes in genes belonging to inflammatory and DAM pathways in microglia, and an increase of TSPO-PET signal in the brain.

One of the fundamental questions in the field is whether microglia are affected by the amyloid plaque environment and if so, whether these changes in cell state are beneficial (*e.g.*, by facilitating inflammation resolution) or pathogenic (*e.g.*, by preventing inflammation resolution or promoting neurotoxicity). To begin to address this question, we profiled methoxy-X04 (+) and (-) microglia derived from the *App*^SAA^ brain using LCMS and assessed impact of Aβ phagocytosis on their lipidome and metabolome. This approach revealed significant alterations that are consistent with cellular dysfunction. One of the most striking manifestations is accumulation of ganglioside GM3 in methoxy-X04 (+) microglia, which might be indicative of a lysosomal dysfunction since gangliosides are glycosphingolipids that typically accumulate in lysosomal storage disorders (LSDs). In fact, lipidomic profiling of sorted microglia from independent LSD models, such as the *Ids* KO model of MPS-II (Hunter syndrome) and a *Grn* KO model of *GRN*-associated frontotemporal dementia, showed similar increases in GM3 [88, 89]. GM3 accumulation has previously been described in the entorhinal cortex of AD patients and the forebrain of AD transgenic mouse models, suggesting that it might be broadly associated with AD pathology [72]. One can speculate that microglial GM3 accumulation may result from phagocytosis of Aβ, which can potentially interfere with normal lysosomal degradative capacity and decrease lysosomal catabolism of gangliosides.

Another prominent phenotype is accumulation of neutral lipids, such as triglycerides and to a lower extent, some CE species. While these lipids are generally found in cytoplasmic lipid droplets where they can store fatty acids and cholesterol while minimizing their lipotoxicity, accumulation of these lipids may also reflect metabolic imbalance and has been associated with pro-inflammatory states and neurotoxicity in other models [10, 22, 90, 91]. Remarkably, these biochemical findings are consistent with Dr. Alzheimer’s original observations of “adipose inclusions” in glial cells neighboring amyloid plaque s[92]. Consistent with these early seminal observations, xenografted human iPSC-derived microglia (xMG) in an AD mouse model background showed similarities with atherosclerotic “foam” macrophages, including increased occurrence of lipid droplets in amyloid plaque-bound xMGs [73]. Taken together with a growing amount of human genetic data linking various genes mediating lipid metabolism to AD (*e.g.*, *APOE4*, *ABCA7*, *CLU*, *TREM2*), these published data as well as lipidomic data from our study lends strong support to the notion dysregulation of microglial lipid metabolism contributes to AD pathogenesis.

One of the most striking observations from our metabolomic study is selective accumulation of the polyamine spermine in methoxy-X04 (+) microglia from the *App*^SAA^ KI mice. Spermine has been shown to modulate various cellular functions, including gene expression, ion channel function, response to oxidative stress, autophagy, metabolism and immune response. Spermine is critical for normal cell function as genetic ablation of spermine synthase (SMS) has been shown to cause oxidative stress and lysosomal impairment [93]. Additionally, spermine binds and neutralizes Aβ [94] and is upregulated in the PSAPP mouse model [95], suggesting that increase in spermine levels in methoxy-X04 (+) microglia may be a compensatory response to prevent the deleterious effects of Aβ. Alternatively, since spermine can be actively transported out of the lysosomes through the lysosome-associated transporter ATP13A2 and can accumulate in these organelles [96], spermine accumulation may reflect lysosomal dysfunction, as speculated for GM3. While our lipidomic and metabolic observations of phagocytic microglia in *App*^SAA^ KI provide evidence of pathway dysregulation, it will be important to assess whether manipulation of these pathways through targeted molecular genetic and/or pharmacological approaches in *App*^SAA^ mice (*e.g.*, *via* targeting GM3, TG/CE, or spermine metabolism) confer benefits or exacerbate specific disease-related phenotypes.

Microglia expression profiles from preclinical mouse models of AD have shown that microglia rewire their transcriptome in a manner that does not fit the canonical M1/M2 macrophage activation paradigm [8, 9, 12, 14, 15, 17–19]. A recent meta-analysis of single-cell RNA-seq studies of microglia from these models has postulated that the microglial diversity observed can be characterized as some combination of five broad states: Homeostatic, DAM-like, IFN-response, MHC-II, and a proliferating state [97]. Expression profiles of microglia from *App*^SAA^ mice show that they exhibit a response similar to what has been seen in 5xFAD mice, with a strong upregulation of the DAM-like signature. Concordant with recent findings in 5xFAD mice reported by Grubman *et al*. [13], transcriptional analysis of methoxy-X04 (+) microglia in *App*^SAA^ mice identified a number of acutely upregulated genes and biological processes involved in the regulation of amyloid beta clearance (*Lrpap1*, *Tnf*, *Trem2*), lipid metabolism (*Apoe*, *Olr1*, *Fabp5*, *Lpl*, *Ch25h*), and lysosomal function (*Gla*, *Galns*, *Ctsd*, *Cst7*). The transcriptional program found in the methoxy-X04 (+) microglia of *App*^SAA^ mice appears to be a hyper-activated version of the methoxy-X04 (-) population when both are compared to microglia from WT mice, which stands in slight contrast to the qualitatively different response between the methoxy-X04 (+) and (-) populations from 5xFAD mice as reported in Grubman et al. [13] (Supplementary Fig. 8c-e).

In addition to neuroinflammation, the *App*^SAA^ mice show elevated levels of biomarkers of neurodegeneration in the CSF. Neurofilament light chain (Nf-L) has received a lot of attention in the last few years and has been studied across various neurological conditions to evaluate the extent of neuronal damage [98]. In AD, Nf-L progressively increases in CSF and plasma and has been correlated with brain atrophy and cognitive decline [99, 100]. Interestingly, we observed a progressive increase of Nf-L levels in the CSF in the *App*^SAA^ mice, starting with trends at 8 months of age. Despite the increase of Nf-L in CSF from control animals with age, we detected a greater separation of Nf-L levels over time, suggesting that neuronal damage increased as pathology worsens in this model. The extent of Nf-L elevation in this model (~1.5-2 fold) was within the range of what has been reported in patients with mild-cognitive impairment and AD [101, 102] and contrasted with the profound elevation of Nf-L levels in CSF reported in the 5xFAD and APP/PS1 mouse models (~10-20 fold) [101, 102], indicating that *App*^SAA^ mice may better recapitulate the rate of neurodegeneration estimated in the human condition than the 5xFAD or APP/PS1 mouse models. The excessive levels of Nf-L increase in those models are likely caused by the accumulation of fAD mutations in APP and PSEN1 in the overexpressed transgenes which result in early and fast accumulation of amyloid plaques. Consistent with the Nf-L increase, we also found that total tau was elevated at 8 months of age, again within range previously reported in patients [103]. As Nf-L levels have been associated with hypometabolism in AD [104], we measured FDG-PET signal in the *App*^SAA^ mice. In contrast to human FDG hypometabolism reported in the later phase of A D[105–109], we observed a cortical FDG hypermetabolism in the *App*^SAA^ mouse model. Similar hypermetabolism was also found in other amyloid models and probably reflects enhanced microglial and astroglial glucose uptake [20, 110]. In addition, it is possible that the neuronal loss in those models is not substantial enough to drive an overall reduction in brain. Despite the mouse to human discrepancy in FDG-PET signal, our data demonstrate increase of biomarkers of neurodegeneration as amyloid pathology accumulates in the *App*^SAA^ mouse model.

We also observed the emergence of behavioral abnormalities in the *App*^SAA^ KI mice, who displayed a robust alteration in spontaneous activities. Locomotor hyperactivity is commonly observed in transgenic mouse models of AD [81]. Although locomotor hyperactivity has been suggested to be linked to APP overexpression in APP/TTA transgenic mice [111], our results indicate that fAD mutations without APP overexpression are sufficient to induce locomotor hyperactivity and habituation deficits. Importantly, we found that the hyperactivity in *App*^SAA^ mice was experience-dependent and emerged as wildtype littermate mice habituate to the environment, particularly during delayed trials, potentially indicating mild impairment in context-dependent habituation, as previously described in other models [84, 112].

To the extent that we could, we included both males and females, different ages and the three genotypes of interest in this study. Early in the project, we made the decision to use wild-type littermate mice as controls. While this experimental approach provides many advantages, we also recognized that the combination of the 3 fAD mutations (Swedish, Arctic and Austrian) in the *App*^SAA^ KI line unfortunately precludes us from determining the relative contribution of each fAD mutation on the generation of Aβ peptides in this model. Generation of humanized Aβ mouse lines with inclusion of a single fAD mutation, and/or dual combination of those mutations (e.g., Swedish and Austrian, Swedish and Arctic, and Arctic and Austrian), would be necessary to rigorously explore the contribution of those mutations on Aβ production and stability. We have ongoing efforts to generate those new lines and will make them also available to the field. A recently generated mouse model expressing humanized Aβ (hAβ-KI) did not form amyloid plaques or show robust immune/glial responses and had only minor signs of neurodegeneration detected at 22 months of age [113]. In addition, a transcriptomics analysis of bulk brain tissue in the hAβ-KI showed that only a very few genes were differentially regulated compared to wild-type control mice, contrasting with the profound gene expression changes reported in microglia from *App*^SAA^ KI mice. Surprisingly, the hAβ-KI mouse model showed behavioral and synaptic deficits after 10-14 months of age, suggesting that humanization of Aβ *per se* may be sufficient to cause minor behavioral abnormalities, at least in the background strain studied (mixed C57BL/6NTac and C57BL/6J background) [113]. At this stage, we thus cannot parse out the potential contribution of Aβ humanization and fAD mutations on behavioral phenotype in the *App*^SAA^ KI mice. Altogether, our observations indicate that biological changes in the *App*^SAA^ KI mouse model were largely driven by the fAD mutations inserted on the *App* gene, which is consistent with the notion that amyloid plaques and associated neurodegeneration are the main pathological driver in *App* KI models.

## Conclusions

**Fig. 8:**
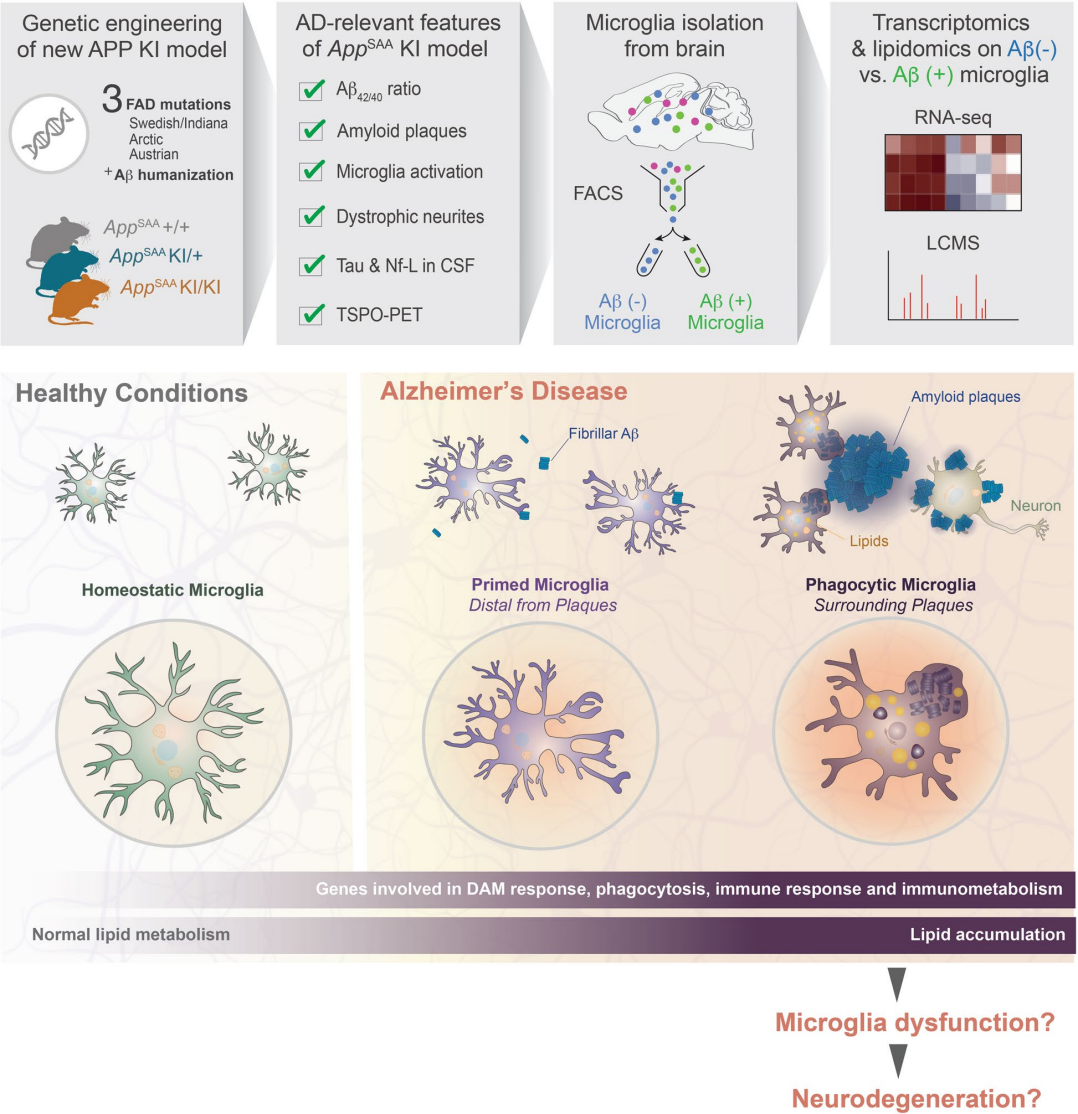
Summary of disease-relevant biology in the *App*^SAA^ mouse model

Our in-depth analysis of the *App*^SAA^ knock-in mouse model confirms emergence of disease-relevant biology and progressive accumulation of pathological hallmarks of AD (Fig. 8). We identified extensive lipid and metabolite alterations in microglia, depending on their stage of phagocytic activity and proximity to amyloid plaques. Based on these findings, this new mouse model provides a helpful tool to investigate multiple relevant aspects of AD biology. We have therefore made it available to the broad scientific community to facilitate AD research and therapeutic development.

## Supplementary Information

**Additional file 1: Supplementary Figure 1.** Levels of Aβ in brain, CSF and plasma from *App*^SAA^ KI mice at 2 months of age. **a** Schematic illustrating the genetic engineering approach of the *App*^SAA^ mouse line. Sp denotes restriction sites for SpeI digestion. **b-e** Aβ40 and Aβ42 concentrations and the corresponding ratio of Aβ42/40 were analyzed from brain insoluble fraction (**b**), brain soluble fraction (**c**), CSF (**d**) and plasma (**e**) from the 3 genotypes of the *App*^SAA^ mouse line at 2-month-old. n = 4 mice per genotype. Graphs are box and whisker plots and P values: one-way ANOVA with Dunnett’s multiple comparison test, each group compared to the *App*^SAA^ +/+ wild-type control group; *P < 0.05, **P < 0.01, and ***P < 0.001.

**Additional file 2: Supplementary Figure 2**. Brain-wide spatio–temporal patterns of plaque deposition in App^SAA^ KI/KI mice recapitulates the pattern seen in human patients with AD. Comparison of relative plaque density in similar structures between human autopsy tissue and four APP mouse models. Human brain regions where Aβ pathology was previously quantified (Thal et al. 2002) are listed in the left column and the corresponding region(s) from the Allen CCFv3 reference atlas are listed in the second column. The median plaque density (plaques per mm3) for each age group and mouse line is indicated by the heatmap in the columns to the right. Cartoons on the right show the anatomical location of the structures that were included in each Phase. The colormap for the plaque density spans from the 10th percentile to the 90th percentile of the plaque density for all structures at the oldest age in each mouse line. Abbreviations: ENTl = entorhinal area, lateral part; ENTm = entorhinal area, medial part, dorsal zone; ACA = anterior cingulate area; RSP = retrosplenial area; LA = lateral amygdalar nucleus; BLA = basolateral amygdalar nucleus; BMA = basomedial amygdalar nucleus; PA = posterior amygdalar nucleus; CEA = central amygdalar nucleus; MEA = medial amygdalar nucleus; COA = cortical amygdalar area; DG = dentate gyrus; PRE = presubiculum; POST = postsubiculum; TH = thalamus; CP = caudoputamen; ACB = nucleus accumbens; HY = hypothalamus; SI = substantia innominata; MA = magnocellular nucleus; NDB = diagonal band nucleus; PAG = periaqueductal gray; SCs = Superior colliculus, sensory related; SCm = superior colliculus, motor related; RN = red nucleus; IO = inferior olivary complex; SNr = substantia nigra, reticular part; SNc = substantia nigra, compact part; GRN = gigantocellular reticular nucleus; IRN = intermediate reticular nucleus; PARN = parvicellular reticular nucleus; CBX = cerebellar cortex; PRNr = pontine reticular nucleus; PRNc = pontine reticular nucleus, caudal part; RAmb = midbrain raphe nuclei; LC = locus ceruleus; PB = parabrachial nucleus; DN = dentate nucleus.

**Additional file 3: Supplementary Figure 3.** Aβ plaque onset and accumulation in *App*^SAA^ KI/KI mice over time. **a** Representative images of brain sections co-stained with anti-amyloid antibody (left) and methoxy-X04 (right) show plaque pathology in *App*^*SAA*^ KI/KI mice from 4-12 months of age. The early plaque deposition in 4-month-old *App*^*SAA*^ KI/KI mice was indicated by white arrowheads. Scale bars = 2 mm. **b** Quantification of brain areas covered by Aβ plaques from 4-month-old to 12-month-old *App*^SAA^ KI/KI mice. N = 3-4 mice per age.

**Additional file 4: Supplementary Figure 4.** Comparison of plaques between 5xFAD mice and *App*^*SAA*^ KI/KI mice at 6 months of age. **a** Representative images of brain sections co-stained with methoxy-X04 and anti-amyloid antibody show plaque pathology in 5xFAD mice and *App*^*SAA*^ KI/KI mice at 6 months of age. Scale bars = 200 μm. **b** Methoxy-X04 and anti-amyloid co-immunostaining reveals lower proportion of methoxy positive plaques in *App*^*SAA*^ KI/KI mice relative to 5xFAD mice at 6-8 months of age. *n*=7-8 mice per genotype. Graphs are box and whisker plots and P values: unpaired t test; ***P < 0.001.

**Additional file 5: Supplementary Figure 5.** Evidence of CAA pathology in *App*^SAA^ mice at 8 and 16 months of age. **a** Representative images of Aβ deposition in *App*^SAA^ +/+, *App*^SAA^ KI/+, and *App*^SAA^ KI/KI mice at 16 months of age; *App*^SAA^ KI/+ and *App*^SAA^ KI/KI exhibit parenchymal Aβ plaque deposition (examples indicated with yellow arrowheads for *App*^SAA^ KI/+) and Aβ deposition associated with leptomeningeal vessels (CAA; examples indicated with white arrows for both *App*^SAA^ KI/+ and *App*^SAA^ KI/KI). Scale bars = 1 mm. **b** Representative confocal images showing accumulation of Aβ surrounding leptomeningeal (pial) vessels (endothelial cells labeled by CD31 and smooth muscle cells labeled by alpha-smooth muscle actin, consistent with putative arteries/arterioles) in *App*^SAA^ mice at 8 and 16 months of age. Scale bars = 50 μm (left images and magnified views). **c-e** Super resolution confocal images from *App*^SAA^ KI/KI mice 16 months of age showing Aβ accumulation in a penetrating parenchymal vessel (**c**), branching leptomeningeal vessel in the ambient cistern (**d**) and a leptomeningeal vessel (**e**), scale bars = 20 μm for images across (**c-e**).

**Additional file 6: Supplementary Figure 6.** Analysis of microglia clustering in *App*^SAA^ mice. **a-d** Histological analysis of microglia and amyloid-β plaques in brain sections from 8-month-old *App*^SAA^ mice. Quantification of hippocampal areas covered by Iba1 (**a**), CD68 (**b**) and the percentage of the CD68 and Iba1 signals overlapping with amyloid-β plaques in the hippocampus (**c**) from *App*^SAA^ KI/KI mice. (**d**) Percentage of amyloid plaque overlapping with CD68 or Iba1 in the hippocampus or cortex. Graphs are box and whisker plots and P values: one-way ANOVA with Dunnett’s multiple comparison test, each group compared to the *App*^SAA^ +/+ control group (*n*=4-6 per group); *P < 0.05 and **P < 0.01.

**Additional file 7: Supplementary Figure 7.** *App*^SAA^ knock-in mice have multiple AD pathology-related changes in astrocytes. **a** Representative images of GFAP and Aβ co-immunolabeling in the CA1 region of the hippocampus (left), and GFAP immunolabeling of entire hemispheres (right) in 18-month-old *App*^SAA^ +/+ and *App*^SAA^ KI/KI mice. **b** Representative images of GLT-1 immunolabeling in the CA1 and CA3 subregions of the hippocampus. **c** Representative images of C3 and GFAP co-immunolabeling in the CA1 subregion of the hippocampus (left) and C3 immunolabeling of entire hemispheres (right) in 18-month-old *App*^SAA^ +/+ and *App*^SAA^ KI/KI mice. **d** Quantification of GFAP immunoreactivity in the CA1, CA3, dentate gyrus (DG), and neocortex (CTX). **e** Quantification of GLT-1 immunoreactivity in the CA1, CA3, DG, and CTX. **f** Quantification of astrocytic C3 immunoreactivity in the CA1, CA3, DG and CTX. Scale bars: 300 μm for hemisphere images and 100 μm for all other images. Graphs show means ± SEM. Mann-Whitney test, *P < 0.05, **P < 0.01 versus *App*^SAA^ +/+; n = 5 *App*^SAA^ +/+, 7 *App*^SAA^ KI/KI.

**Additional file 8: Supplementary Figure 8.** Comparative analysis of microglia transcriptome. **a** Absolute expression (log2 counts per million) of cell type specific markers shows strong enrichment and purity of microglia cell population analyzed in this Study. **b** Log2 fold change of methoxy-X04 (-) *App*^SAA^ +/+ microglia vs methoxy-X04 (-) WT microglia (x-axis) vs log2 fold change of methoxy-X04 (+) *App*^SAA^ +/+ microglia vs methoxy-X04 (-) WT (y-axis). Only genes with log2 fold change >= 1.2 and FDR <= 10% in either comparison are shown. Genes from the homeostatic (light blue), DAM (fuscia), and PIGs (dark blue) signatures are highlighted. **c** Same as in (b) but data taken from bulk RNA-seq data from Grubman et al. Genes highlighted in green are the methoxy (+) signature genes identified in scRNAseq data from Grubman et al. (supplemental table 5: specific X04+ DEGs) with log2FC >= 1. **d** comparison of log2 fold changes of genes identified in *App*^SAA^ KI/KI methoxy-X04 (+) microglia vs WT (x-axis) and 5xFAD methoxy-X04 (+) microglia vs WT (y-axis) in Grubman et al. Spearman’s rho: 0.66, *p*-value < 1e-10. **e** Functional enrichment scores of the union of top 10 Gene Ontology Biological Process categories identified in methoxy (+) microglia vs WT in *App*^SAA^ KI/KI mice (orange) and 5xFAD mice from Grubman et al. (purple). Bars indicate -log10(q-value) from an overrepresentation analysis of upregulated genes in each of the ontology terms.

**Additional file 9: Table s1.** Primers.

**Additional file 10: Table s2.** Mouse Cohorts.

**Additional file 11: Table s3.** Plaque Count Whole-Bean

**Additional file 12: Table s4.** Gene Sets in Figure 3 and Figure 4.

**Additional file 13: Table s5.** microglia baseline abundance.

**Additional file 14: Table s6.** microglia diff abundance analysis.

**Additional file 15: Table s7.** methoxyx04_baseline_abundance.

**Additional file 16: Table s8.** methoxyx04_diff_abundance analysis

**Additional file 17: Table s9.** Summary of behavioral results at the Jackson Research Laboratory.

## Abbreviations

Aβ: Amyloid-β protein
AD: Alzheimer’s disease
APP: Amyloid precursor protein
ANOVA: analysis of variance
CAA: cerebral amyloid angiopathy
CE: cholesteryl esters
CSF: Cerebrospinal fluid
DAM: Disease associated microglia
DEGs: Differentially expressed genes
KI: Knock-in
LCMS: liquid chromatography and mass spectrometry
PIG: Plaque-induced genes
PSEN: presenilin 1
Nf-L: Neurofilament light chain
SEM: Standard error of the mean
TG: triglycerides
WT: wild type
