# Supplementary figures and images for: Novel App knock-in mouse model shows key features of amyloid pathology and reveals profound metabolic dysregulation of microglia
Source: Mol Neurodegener. 2022 Jun 11;17:41. doi: 10.1186/s13024-022-00547-7 (PMC9188195; doi:10.1186/s13024-022-00547-7)

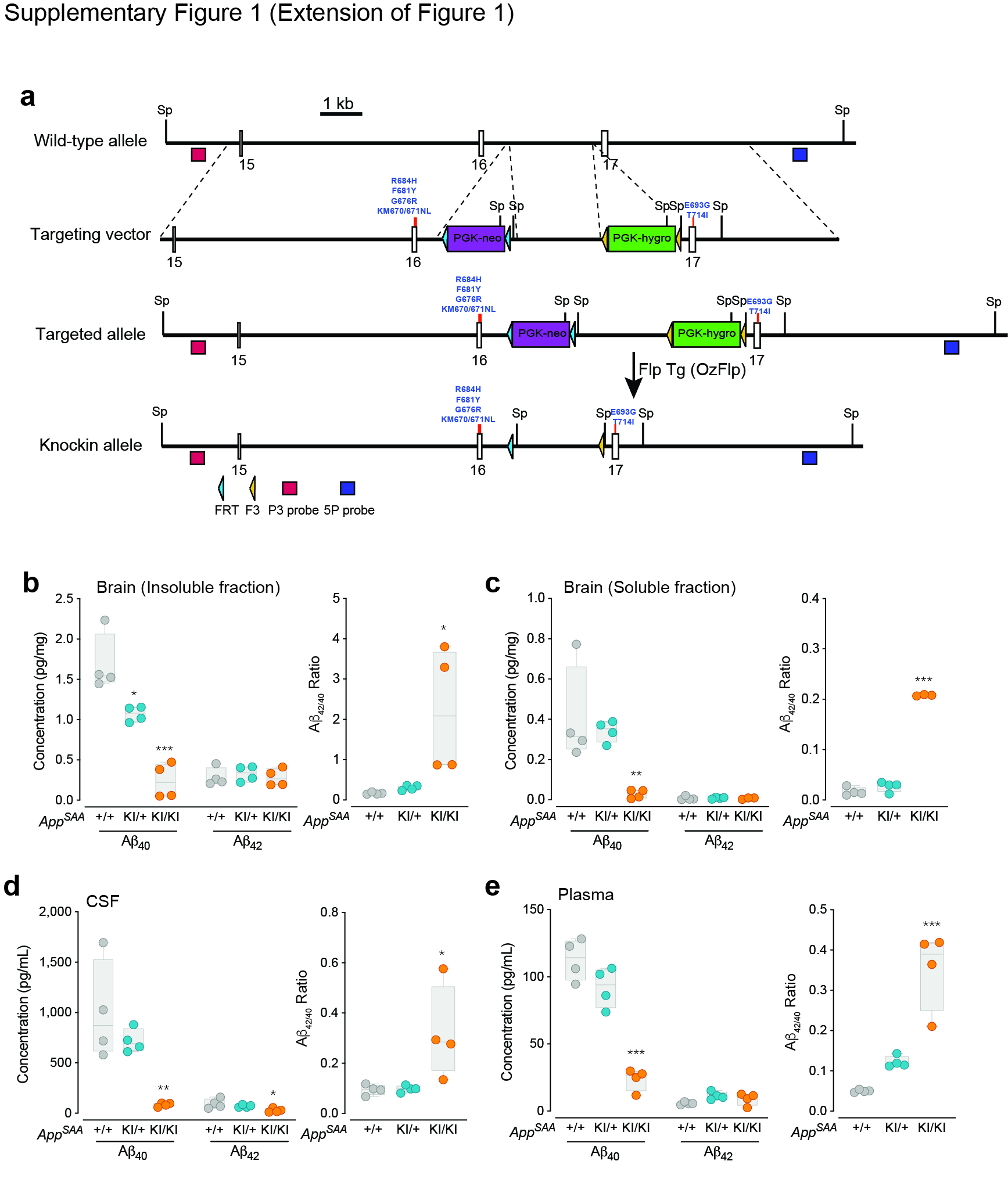

Supplement: Supplementary file 1 — Additional file 1: Supplementary Figure 1. Levels of Aβ in brain, CSF and plasma from AppSAA KI mice at 2 months of age. a Schematic illustrating the genetic engineering approach of the AppSAA mouse line. Sp denotes restriction sites for SpeI digestion. b-e Aβ40 and Aβ42 concentrations and the corresponding ratio of Aβ42/40 were analyzed from brain insoluble fraction (b), brain soluble fraction (c), CSF (d) and plasma (e) from the 3 genotypes of the AppSAA mouse line at 2-month-old. n = 4 mice per genotype. Graphs are box and whisker plots and P values: one-way ANOVA with Dunnett’s multiple comparison test, each group compared to the AppSAA +/+ wild-type control group; *P < 0.05, **P < 0.01, and ***P < 0.001. [file 13024_2022_547_MOESM1_ESM.jpg]

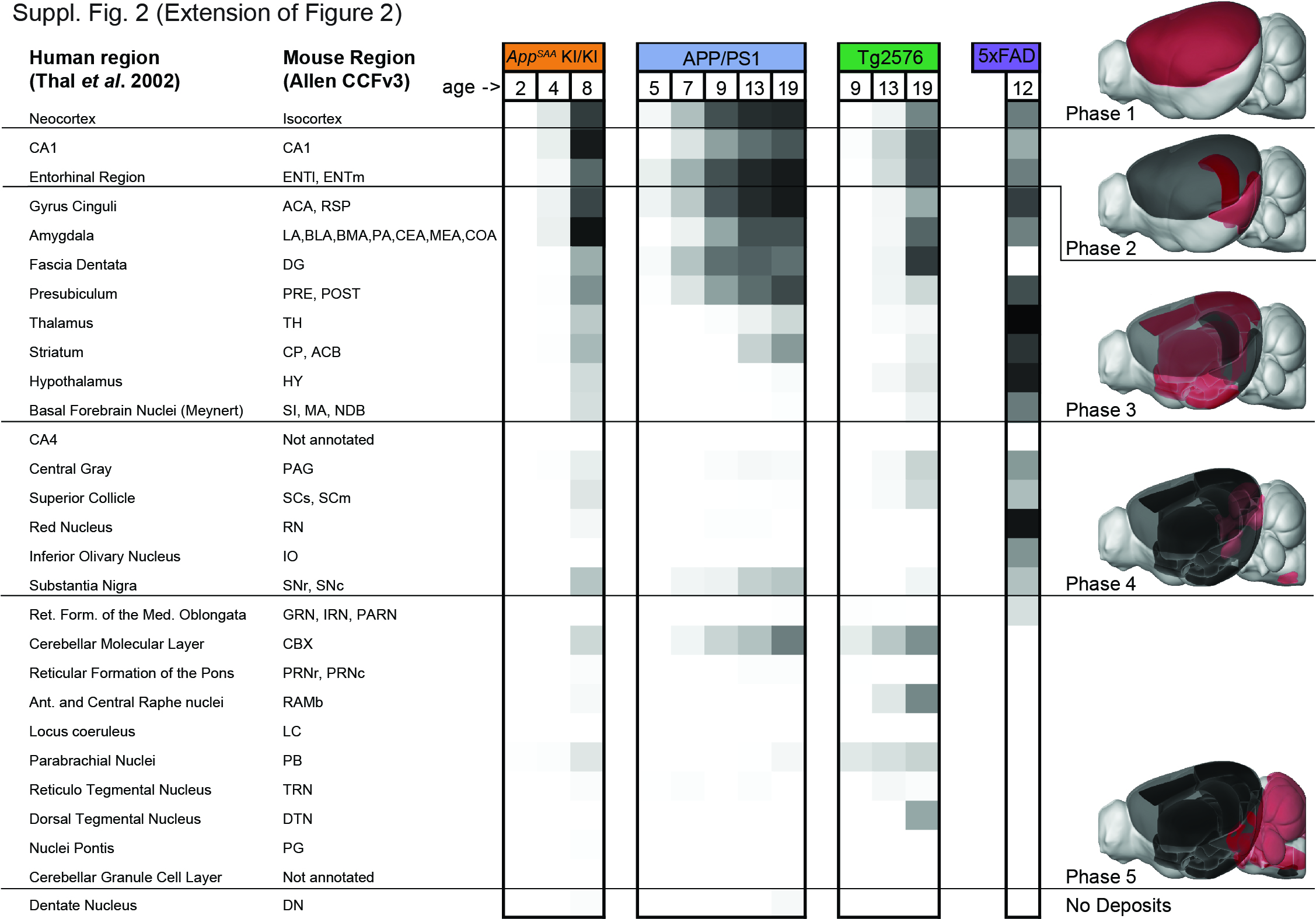

Supplement: Supplementary file 2 — Additional file 2: Supplementary Figure 2. Brain-wide spatio–temporal patterns of plaque deposition in AppSAA KI/KI mice recapitulates the pattern seen in human patients with AD. Comparison of relative plaque density in similar structures between human autopsy tissue and four APP mouse models. Human brain regions where Aβ pathology was previously quantified (Thal et al. 2002) are listed in the left column and the corresponding region(s) from the Allen CCFv3 reference atlas are listed in the second column. The median plaque density (plaques per mm3) for each age group and mouse line is indicated by the heatmap in the columns to the right. Cartoons on the right show the anatomical location of the structures that were included in each Phase. The colormap for the plaque density spans from the 10th percentile to the 90th percentile of the plaque density for all structures at the oldest age in each mouse line. Abbreviations: ENTl = entorhinal area, lateral part; ENTm = entorhinal area, medial part, dorsal zone; ACA = anterior cingulate area; RSP = retrosplenial area; LA = lateral amygdalar nucleus; BLA = basolateral amygdalar nucleus; BMA = basomedial amygdalar nucleus; PA = posterior amygdalar nucleus; CEA = central amygdalar nucleus; MEA = medial amygdalar nucleus; COA = cortical amygdalar area; DG = dentate gyrus; PRE = presubiculum; POST = postsubiculum; TH = thalamus; CP = caudoputamen; ACB = nucleus accumbens; HY = hypothalamus; SI = substantia innominata; MA = magnocellular nucleus; NDB = diagonal band nucleus; PAG = periaqueductal gray; SCs = Superior colliculus, sensory related; SCm = superior colliculus, motor related; RN = red nucleus; IO = inferior olivary complex; SNr = substantia nigra, reticular part; SNc = substantia nigra, compact part; GRN = gigantocellular reticular nucleus; IRN = intermediate reticular nucleus; PARN = parvicellular reticular nucleus; CBX = cerebellar cortex; PRNr = pontine reticular nucleus; PRNc = pontine reticular nucleus, caudal par [file 13024_2022_547_MOESM2_ESM.jpg]

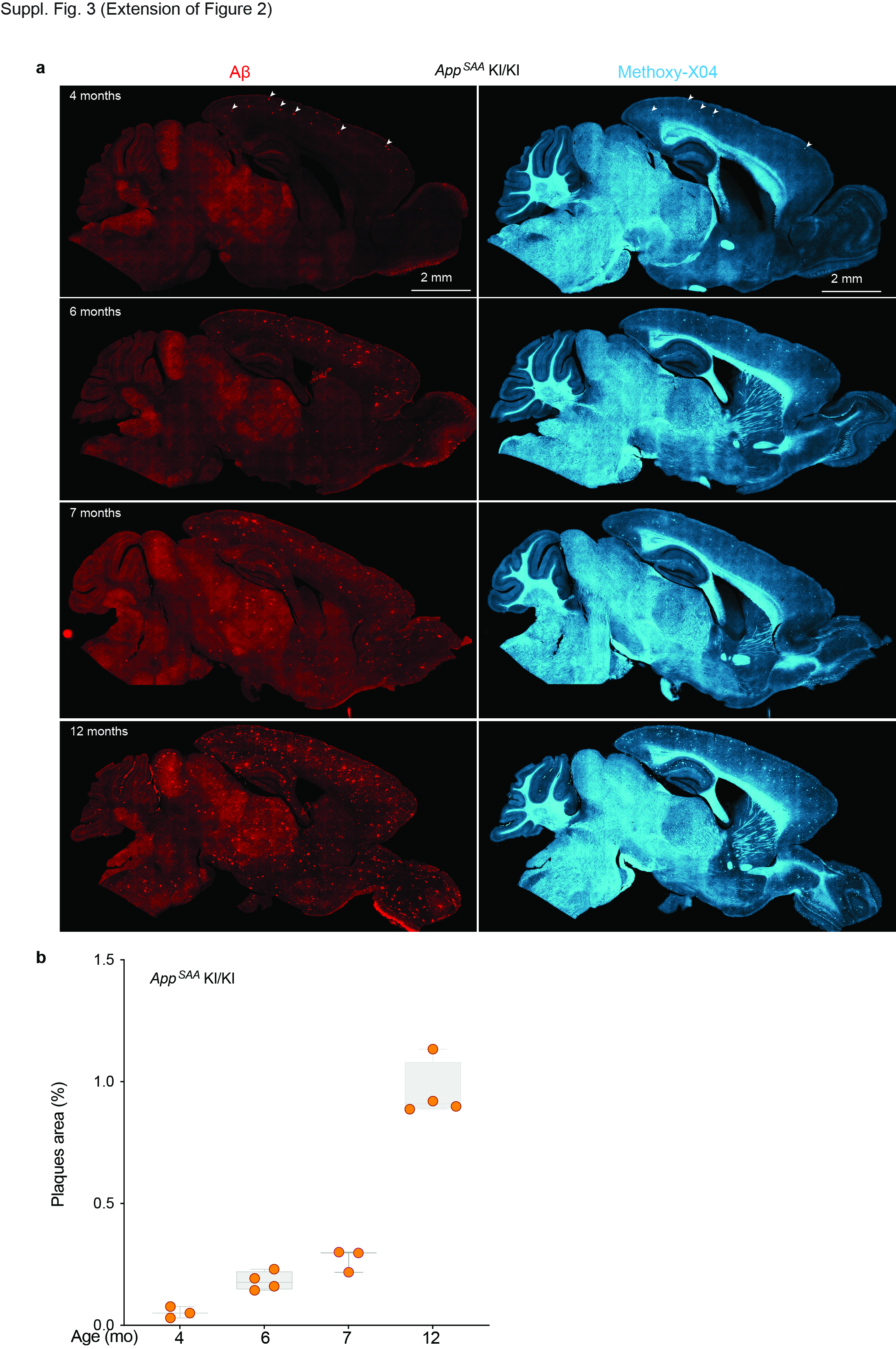

Supplement: Supplementary file 3 — Additional file 3: Supplementary Figure 3. Aβ plaque onset and accumulation in AppSAA KI/KI mice over time. a Representative images of brain sections co-stained with anti-amyloid antibody (left) and methoxy-X04 (right) show plaque pathology in AppSAA KI/KI mice from 4-12 months of age. The early plaque deposition in 4-month-old AppSAA KI/KI mice was indicated by white arrowheads. Scale bars = 2 mm. b Quantification of brain areas covered by Aβ plaques from 4-month-old to 12-month-old AppSAA KI/KI mice. N = 3-4 mice per age. [file 13024_2022_547_MOESM3_ESM.jpg]

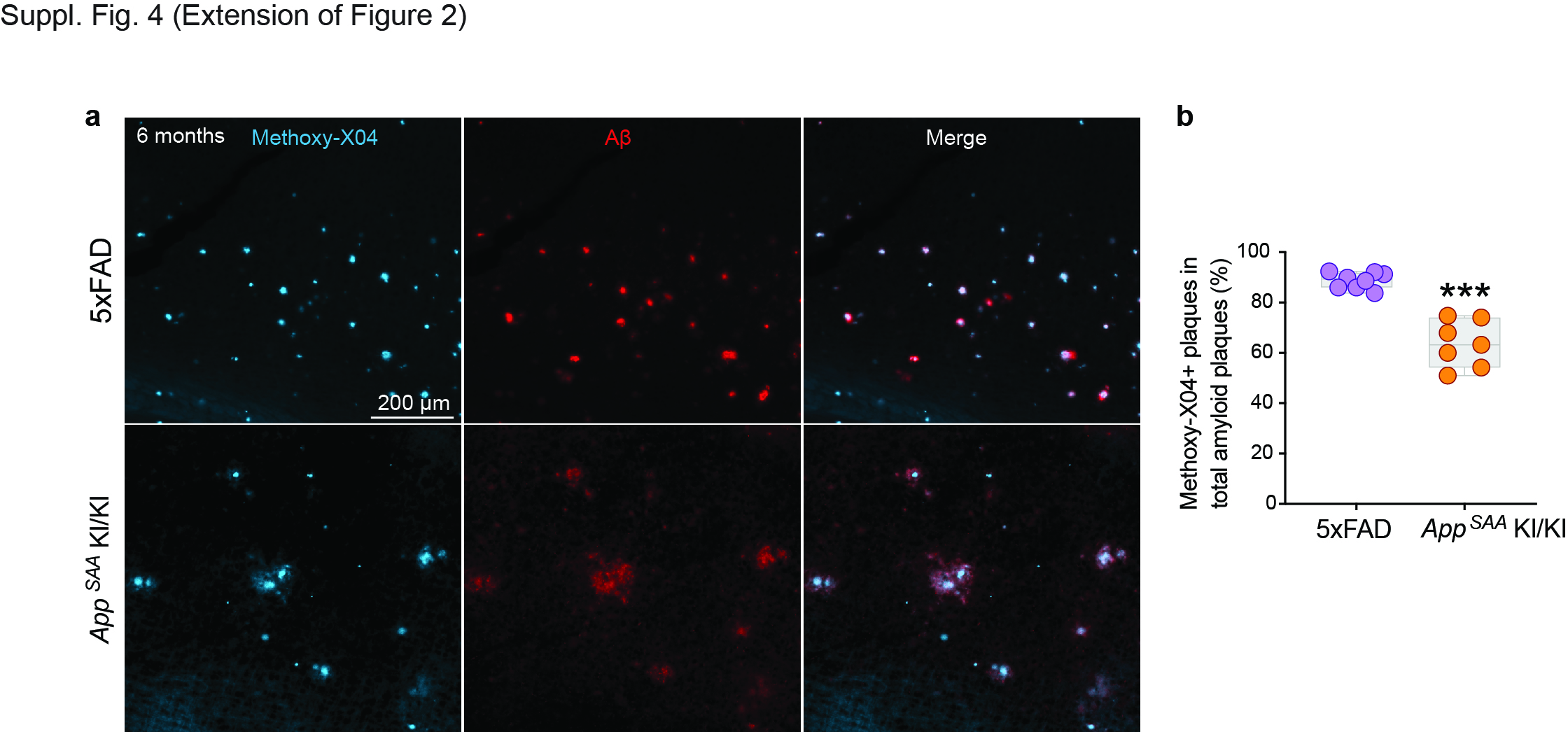

Supplement: Supplementary file 4 — Additional file 4: Supplementary Figure 4. Comparison of plaques between 5xFAD mice and AppSAA KI/KI mice at 6 months of age. a Representative images of brain sections co-stained with methoxy-X04 and anti-amyloid antibody show plaque pathology in 5xFAD mice and AppSAA KI/KI mice at 6 months of age. Scale bars = 200 μm. b Methoxy-X04 and anti-amyloid co-immunostaining reveals lower proportion of methoxy positive plaques in AppSAA KI/KI mice relative to 5xFAD mice at 6-8 months of age. n=7-8 mice per genotype. Graphs are box and whisker plots and P values: unpaired t test; ***P < 0.001. [file 13024_2022_547_MOESM4_ESM.jpg]

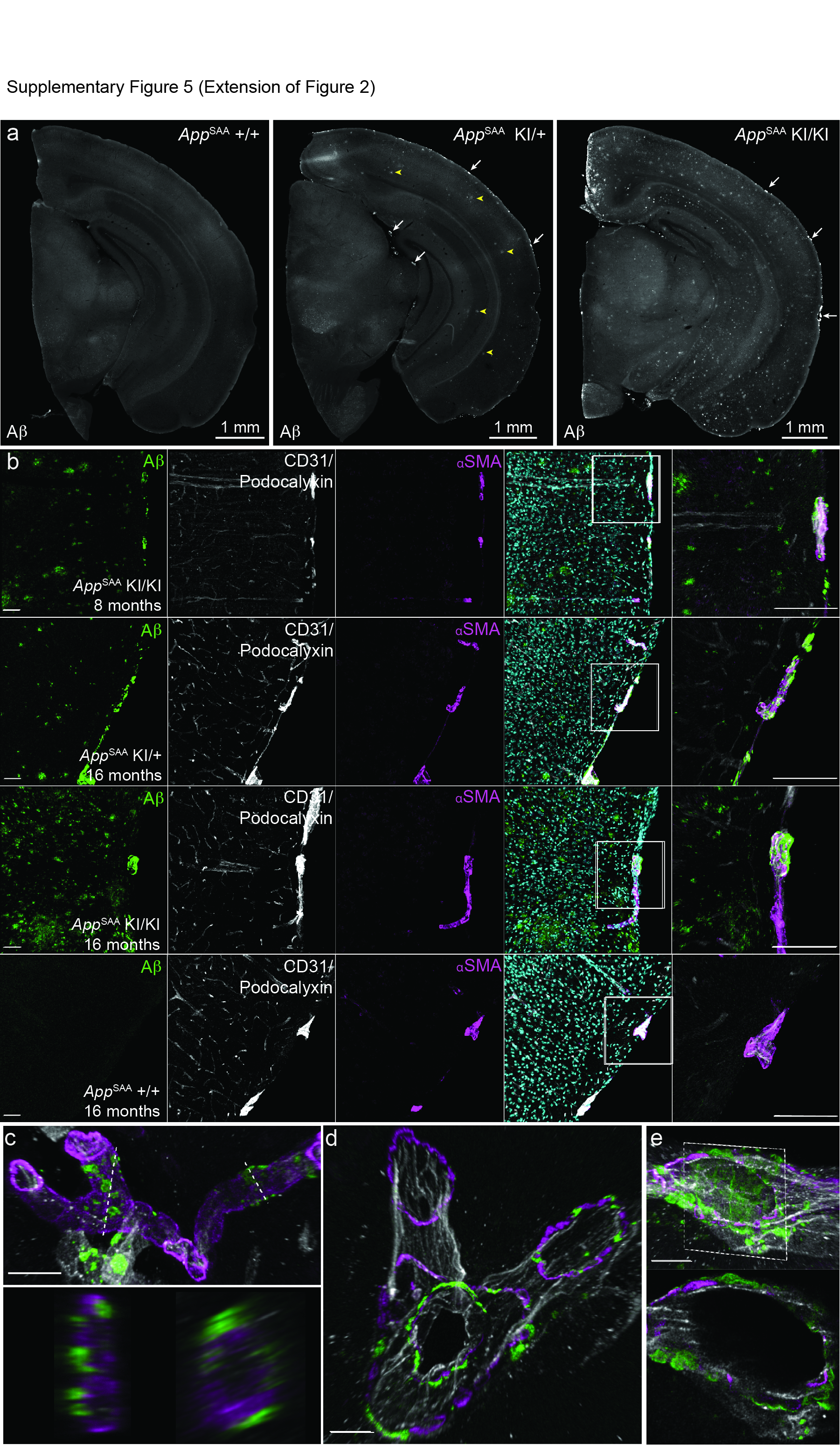

Supplement: Supplementary file 5 — Additional file 5: Supplementary Figure 5. Evidence of CAA pathology in AppSAA mice at 8 and 16 months of age. a Representative images of Aβ deposition in AppSAA +/+, AppSAA KI/+, and AppSAA KI/KI mice at 16 months of age; AppSAA KI/+ and AppSAA KI/KI exhibit parenchymal Aβ plaque deposition (examples indicated with yellow arrowheads for AppSAA KI/+) and Aβ deposition associated with leptomeningeal vessels (CAA; examples indicated with white arrows for both AppSAA KI/+ and AppSAA KI/KI). Scale bars = 1 mm. b Representative confocal images showing accumulation of Aβ surrounding leptomeningeal (pial) vessels (endothelial cells labeled by CD31 and smooth muscle cells labeled by alpha-smooth muscle actin, consistent with putative arteries/arterioles) in AppSAA mice at 8 and 16 months of age. Scale bars = 50 μm (left images and magnified views). c-e Super resolution confocal images from AppSAA KI/KI mice 16 months of age showing Aβ accumulation in a penetrating parenchymal vessel (c), branching leptomeningeal vessel in the ambient cistern (d) and a leptomeningeal vessel (e), scale bars = 20 μm for images across (c-e). [file 13024_2022_547_MOESM5_ESM.jpg]

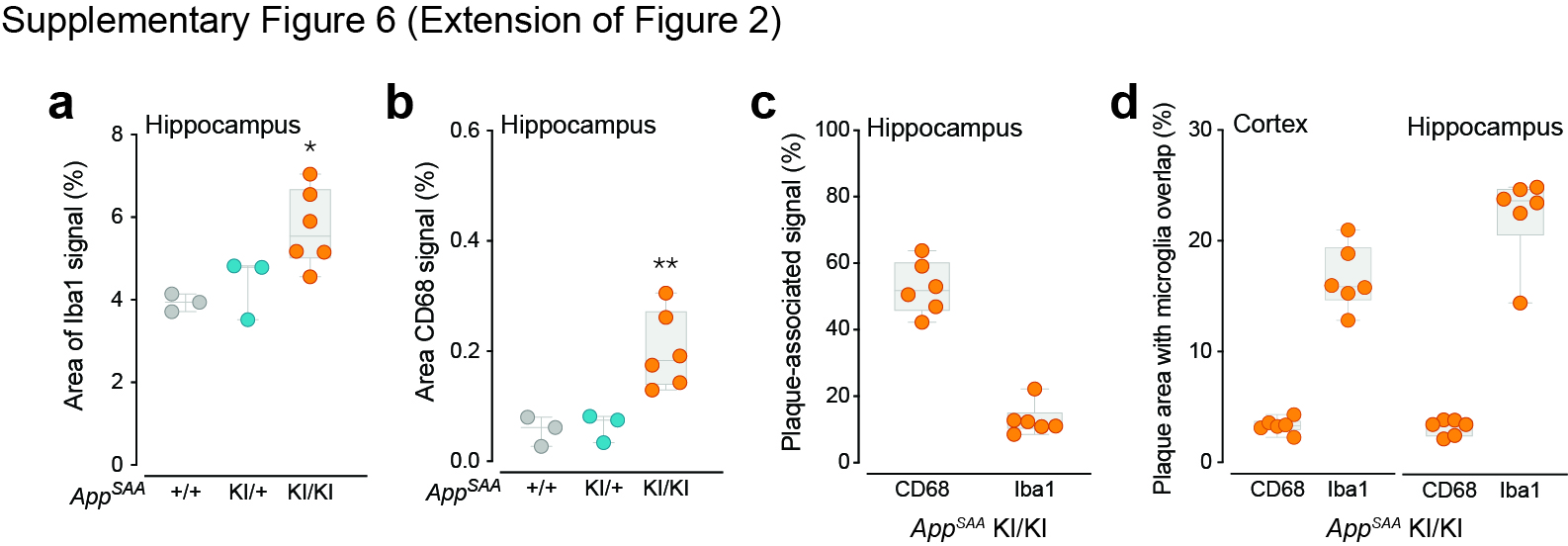

Supplement: Supplementary file 6 — Additional file 6: Supplementary Figure 6. Analysis of microglia clustering in AppSAA mice. a-d Histological analysis of microglia and amyloid-β plaques in brain sections from 8-month-old AppSAA mice. Quantification of hippocampal areas covered by Iba1 (a), CD68 (b) and the percentage of the CD68 and Iba1 signals overlapping with amyloid-β plaques in the hippocampus (c) from AppSAA KI/KI mice. (d) Percentage of amyloid plaque overlapping with CD68 or Iba1 in the hippocampus or cortex. Graphs are box and whisker plots and P values: one-way ANOVA with Dunnett’s multiple comparison test, each group compared to the AppSAA +/+ control group (n=4-6 per group); *P < 0.05 and **P < 0.01. [file 13024_2022_547_MOESM6_ESM.jpg]

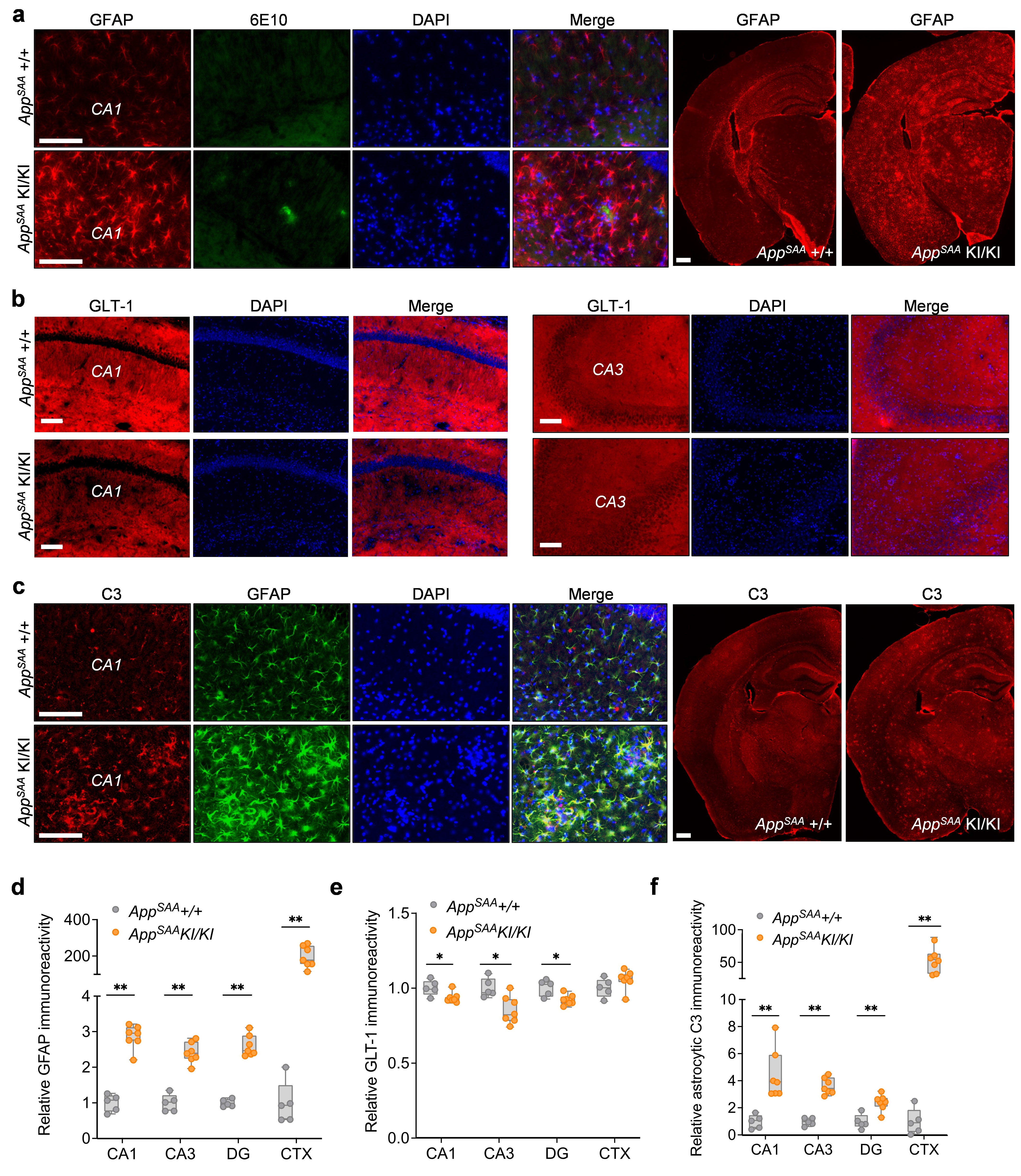

Supplement: Supplementary file 7 — Additional file 7: Supplementary Figure 7. AppSAA knock-in mice have multiple AD pathology-related changes in astrocytes. a Representative images of GFAP and Aβ co-immunolabeling in the CA1 region of the hippocampus (left), and GFAP immunolabeling of entire hemispheres (right) in 18-month-old AppSAA +/+ and AppSAA KI/KI mice. b Representative images of GLT-1 immunolabeling in the CA1 and CA3 subregions of the hippocampus. c Representative images of C3 and GFAP co-immunolabeling in the CA1 subregion of the hippocampus (left) and C3 immunolabeling of entire hemispheres (right) in 18-month-old AppSAA +/+ and AppSAA KI/KI mice. d Quantification of GFAP immunoreactivity in the CA1, CA3, dentate gyrus (DG), and neocortex (CTX). e Quantification of GLT-1 immunoreactivity in the CA1, CA3, DG, and CTX. f Quantification of astrocytic C3 immunoreactivity in the CA1, CA3, DG and CTX. Scale bars: 300 μm for hemisphere images and 100 μm for all other images. Graphs show means ± SEM. Mann-Whitney test, *P < 0.05, **P < 0.01 versus AppSAA +/+; n = 5 AppSAA +/+, 7 AppSAA KI/KI. [file 13024_2022_547_MOESM7_ESM.jpg]

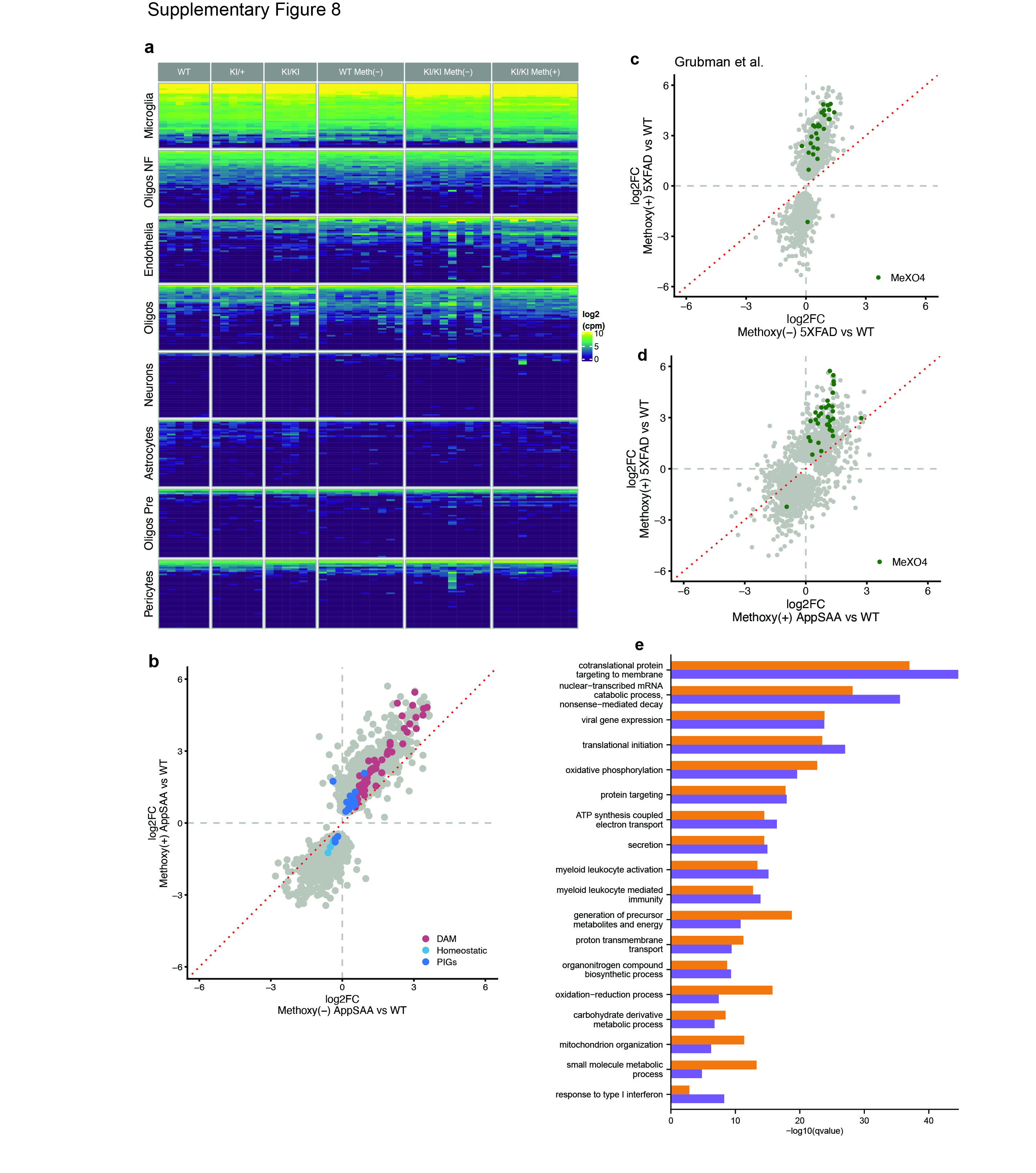

Supplement: Supplementary file 8 — Additional file 8: Supplementary Figure 8. Comparative analysis of microglia transcriptome. a Absolute expression (log2 counts per million) of cell type specific markers shows strong enrichment and purity of microglia cell population analyzed in this Study. b Log2 fold change of methoxy-X04 (-) AppSAA +/+ microglia vs methoxy-X04 (-) WT microglia (x-axis) vs log2 fold change of methoxy-X04 (+) AppSAA +/+ microglia vs methoxy-X04 (-) WT (y-axis). Only genes with log2 fold change >= 1.2 and FDR <= 10% in either comparison are shown. Genes from the homeostatic (light blue), DAM (fuscia), and PIGs (dark blue) signatures are highlighted. c Same as in (b) but data taken from bulk RNA-seq data from Grubman et al. Genes highlighted in green are the methoxy (+) signature genes identified in scRNAseq data from Grubman et al. (supplemental table 5: specific X04+ DEGs) with log2FC >= 1. d comparison of log2 fold changes of genes identified in AppSAA KI/KI methoxy-X04 (+) microglia vs WT (x-axis) and 5xFAD methoxy-X04 (+) microglia vs WT (y-axis) in Grubman et al. Spearman’s rho: 0.66, p-value < 1e-10. e Functional enrichment scores of the union of top 10 Gene Ontology Biological Process categories identified in methoxy (+) microglia vs WT in AppSAA KI/KI mice (orange) and 5xFAD mice from Grubman et al. (purple). Bars indicate -log10(q-value) from an overrepresentation analysis of upregulated genes in each of the ontology terms. [file 13024_2022_547_MOESM8_ESM.jpg]
