# Supplementary material for: Novel App knock-in mouse model shows key features of amyloid pathology and reveals profound metabolic dysregulation of microglia
Source: Mol Neurodegener. 2022 Jun 11;17:41. doi: 10.1186/s13024-022-00547-7 (PMC9188195; doi:10.1186/s13024-022-00547-7)
Supplement: Supplementary file 9 — Additional file 9: Table s1. Primers. [file 13024_2022_547_MOESM9_ESM.docx]

**Supplementary Table 1. Primer sequence and assays for mouse model generation**

| P1915_41 | CTAATACTGACATGAATGAGGTCTGCTC |
| --- | --- |
| P1915_51 | TAAGCATTGGTAAGACGTCTATATGCTGGACTTCTTTCTGCCC |
| P1915_74 | TAAGCATTGGTAAACCGGTAGGCGCCAACCGGCTCCGTTCTTTGGTGGCCCCTTCGCGCCACCTTCTACTCCT  CCCCTAGTCAG |
| P1915_53 | CTAAGGCGCGCCGAAGTTCCTATACTATTTGAAGAATAGGAACTTCGATCTATAGATCATGAGTGGGAGGAATG  AGCTGGCCCTTAATTTG |
| P1915_46 | TAAGCATTGGTAAACGCGTCATGCCATAATTAAAAGGGGAGGG |
| P1915_56 | CTAAATTGAGTAGCTGTAGGAGGAGGTA |
| 5’probe for southern blotting | GGCATCTCAATTCCCTGTCTTTTCTTGGTTTGCTTTGCGTGCTAATCCTCCATGCCCTTTGCTGCTGGGAAGC  AGAAGGGGTTCACGATTCCTGCCATGGGGACAGCTTAGCCCTCAGTGTTCACAGGGCTCTGGGCTTCCGAA  TAGATTGCTTTTAAAGGCACCCGAGGAGAAGAGTCCAAATGGCATCTTTTAATCCCCAATCGTCTTGAATTCT  GGAAGTGAATATTCATGTCTGGGGAGAAACCTCCGGCTAAACAAAGAACAAGGATGAGCCTTCCTCCTAGCA  AGGGCCCTCAAAGCTACCTCGAGGACTGTGCCAATCCCTCTGTGACCACTGTGTGCCTCTAGCAACAGACTG  AAATTGTGGG |
| 3’probe for southern blotting | CCTTGTCAGAAAACAACTTCCCTCAGCTCAGACTGGATTTTGAAGAAATAAGGAAGGGAGAGAGAGGGCAAC  CAAGCTTTAAGGATTAACAAGTGGCTGAAGGGCAGATGAAGGCAAGGGAGGAGGAAGTCCCCTGGGTCAGG  TCTGGCAAGTGTAAAAGAGAGAAAGAAATCTGAGCGTAGTGGTGGAGGGAAGCCAGCGGGAGTTTGATGGG  AGCCTTGGGGACCCACAGTTTCAACCATTTAGCTTACAGAGGCTCACAGGCCAAGTGGTCGAAAGTGACCAC  AACCCCAAATCCGTTCTGAATGGATGGCACAGTTGATAAGAATGG |
| Hygro1 probe for southern blotting | CTGTCGAGAAGTTTCTGATCGAAAAGTTCGACAGCGTCTCCGACCTGATGCAGCTCTCGGAGGGCGAAGAAT  CTCGTGCTTTCAGCTTCGATGTAGGAGGGCGTGGATATGTCCTGCGGGTAAATAGCTGCGCCGATGGTTTCT  ACAAAGATCGTTATGTTTATCGGCACTTTGCATCGGCCGCGCTCCCGATTCCGGAAGTGCTTGACATTGGGGA  ATTCAGCGAGAGCCTGACCTATTGCATCTCCCGCCGTGCACAGGGTGTCACGTTGCAAGACCTGCCTGAAAC  CGAACTGCCCGCTGTTCTGCAGCCGGTCGCGGAGGCCATGGATGCGATCGCTGCGGCCGATCTTAGCCAGA  CGAGCGGGTTCGGCCCATTCGGACCGCAAGGAATCGGTCAATACACTACATGGCGTGATTTCATATGCGCGA  TTGCTGATCCCCATGTGTATCACTGGCAAACTGTGATGGACGACACCGTCAGTGCGTCCGTCGCGCAGGCTC  TCGATGAGCTGATGCTTTGGGCCGAGGACTGCCCCGAAGTCCGGCACCTCGTGCACGCGGATTTCGGCTCCA  ACAATGTCCTGACGGACAATGGCCGCATAACAGCGGTCATTGACTGGAGCGAGGCGATGTTCGGGGATTCCC  AATACGAGGTCGCCAACATCTTCTTCTGGAGGCCGTGGTTGGCTTGTATGGAGCAGCAGACGCGCTACTTCGA  GCGGAGGCATCCGGAGCTTGCAGGATCGCCGCGGCTCCGGGCGTATATGCTCCGCATTGGTCTTGACCAACT  CTATCAGAGCTTGGTTGACGGCAATTTCGATGATGCAGCTTGGGCGCAGGGTCGATGCGACGCAATCGTCCGA  TCCGGAGCCGGGACTGTCGGGCGTACACAAATCGCCCGCAGAAGCGCGGCCGTCTGGACCGATGGCTGTGT  AGAAGTACTCG |
| enP probe for southern blotting | TAAGCATTGGTAAAGAGCACATTTGTTATGTAAGTTAGTGCCAACAGCTCCCTAAATAATTCTTTCCAGGCGGCT  TCAAGATAGGTGAATGACCGATTTCCATCGCTAAATCCATCCCTGCTGCAGTTTGCAAAGGCGAGGTAAACTAG  AGCAGATAAAATTTTTCTAAGTGGATGTTACTCACAGCGCATCTGCGGCAAAATTAGAAATGGCTCGTAATTAAT  CCCCTCGCCAACCAAAAATGCTAGCTCAACACATTTATTAAAGGAGATCATTAATTAACTAATGATACTACAGTG  AGGTGATCGGGAATAAATTTAG |
| neo probe for southern blotting | TAAGCATTGGTAAGACTGGGCACAACAGACAATCGGCTGCTCTGATGCCGCCGTGTTCCGGCTGTCAGCGCAG  GGGCGCCCGGTTCTTTTTGTCAAGACCGACCTGTCCGGTGCCCTGAATGAACTGCAGGACGAGGCAGCGCGGC  TATCGTGGCTGGCCACGACGGGCGTTCCTTGCGCAGCTGTGCTCGACGTTGTCACTGAAGCGGGAAGGGACTG  GCTGCTATTGGGCGAAGTGCCGGGGCAGGATCTCCTGTCATCTCACCTTGCTCCTGCCGAGAAAGTATCCATCA  TGGCTGATGCAATGCGGCGGCTGCATACGCTTGATCCGGCTACCTGCCCATTCGACCACCAAGCGAAACATCGC  ATCGAGCGAGCACGTACTCGGATGGAAGCCGGTCTTGTCGATCAGGATGATCTGGACGAAGAGCATCAGGGGCT  CGCGCCAGCCGAACTGTTCGCCAGGCTCAAGGCGCGCATGCCCGACGGCGATGATCTCGTCGTGACCCATGGC  GATGCCTGCTTGCCGAATATCATGGTGGAAAATGGCCGCTTTTCTGGATTCATCGACTGTGGCCGGCTGGGTGTG  GCGGACCGCTATCAGGACATAGCGTTGGCTACCCGTGATATTGCTGAAGAGCTTGGTTAG |
| qPCR for ES cell screening | 1915_Lo5WT assay (Ozgene) |
| qPCR to confirm 5’targeting | 1915_Lo5WT assay (Ozgene) |
| qPCR to confirm 3’targeting | 1915_LoWT3 assay (Ozgene) |
| qPCR to confirm absence of random integration: | 1638_goNoz assay (Ozgene) |
| qPCR to confirm the copy numbers of the Y Chromosome in the ES cells | 1638_LoChrY assay (Ozgene) |
| qPCR to confirms the copy numbers of the Chromosome 8 in the ES cells | 1638_goChr8 assay (Ozgene) |
