# Supplementary material for: Novel App knock-in mouse model shows key features of amyloid pathology and reveals profound metabolic dysregulation of microglia
Source: Mol Neurodegener. 2022 Jun 11;17:41. doi: 10.1186/s13024-022-00547-7 (PMC9188195; doi:10.1186/s13024-022-00547-7)
Supplement: Supplementary file 10 — Additional file 10: Table s2. Mouse Cohorts. [file 13024_2022_547_MOESM10_ESM.docx]

**Supplementary Table 2. Animal cohorts used for each figure panel**

| **Mouse Cohort** | **Age (months)** | **Sample size and sex by genotype**  (M: males, F: females) | | | **Corresponding figure panels** |
| --- | --- | --- | --- | --- | --- |
|  |  | ***App*^SAA^ +/+** | ***App*^SAA^ KI/+** | ***App*^SAA^ KI/KI** |  |
| 1 | 2 | 2M/ 2F | 3M/ 1F | 1M/ 3F | - Fig. 1a-b - Suppl. Fig. 1b-e |
| 2 | 4 | 4M/ 2F | 4M/ 2F | 4M/ 2F | - Fig. 1c-d |
| 3 | 8 | 3M/ 3F | 2M/ 4F | 4M/ 2F | - Fig. 2d, e, f, g, j, k, l - Fig. 3a-e - Suppl. Fig. 5b - Suppl. Fig. 6 - Suppl. Fig. 8a |
| 4 | 2 | 1M/ 1F | 1M | 4M/ 1F | - Fig. 2a-c, 2h, 2i - Suppl. Fig. 2 - Fig. 4a-b |
|  | 4 | 2F | 6M | 3M/ 3F |  |
|  | 8 | 1M/ 1F | 3M/ 3F | 7M/ 4F |  |
| 5 | 6-8 | - | - | 3M/ 4F | - Suppl. Fig. 3 - Suppl. Fig. 4 |
| 6 | 8 | 3M/ 7F | - | 8M/ 2F | - Fig. 2m, n - Fig. 4 c-i - Fig. 5a-c - Suppl. Fig. 8a, b, d, e |
| 7 | 16 | 1M/3F | 1M/1F | 3M | - Suppl. Fig. 5a-e |
| 8 | 2 | 3M/ 2F | - | 2M/ 3F | - Fig. 2i |
|  | 8 | 3M/ 4F | - | 7M/ 5F |  |
|  | 12 | 6M/ 5F | - | 6M/ 7F |  |
|  | 18 | 18M/ 15F | - | 16M/ 14F |  |
|  | 23 | 2M/ 2F | - | 3M/ 2F |  |
| 9 | 5 | 3M/ 5F | - | 5M/ 3F | - Fig. 6 |
|  | 9 | 4F | - | 3M |  |
|  | 12 | 2M/ 13F | - | 3M/ 2F |  |
|  | 20 | 5M/ 3F | - | 3M/ 2F |  |
| 10 | 4 | 15M/ 15F | 15M/ 15F | 15M/ 15F | - Fig. 7a-c - Suppl. Fig. 3 - Suppl. Fig. 7 |
|  | 8 | 15M/ 15F | 15M/ 15F | 15M/ 13F |  |
|  | 12 | 15M/ 15F | 15M/ 14F | 15M/ 13F |  |
|  | 18 | 15M/ 15F | 15M/ 14F | 15M/ 11F |  |
| 11 | 16-17 | 14M/ 12F | - | 11M/ 10F | - Fig. 7d |
