# Supplementary material for: Novel App knock-in mouse model shows key features of amyloid pathology and reveals profound metabolic dysregulation of microglia
Source: Mol Neurodegener. 2022 Jun 11;17:41. doi: 10.1186/s13024-022-00547-7 (PMC9188195; doi:10.1186/s13024-022-00547-7)
Supplement: Supplementary file 17 — Additional file 17: Table s9. Summary of behavioral results at the Jackson Research Laboratory. [file 13024_2022_547_MOESM17_ESM.docx]

| Assay | Measure | 4-month-old | 8-month-old | 12-month-old | 18-month-old |
| --- | --- | --- | --- | --- | --- |
| Frailty test | Score | M: 8.108% +/-0.1459 (p=0.5834)  F:-11.233% +/- .1611 (p=0.4913) | M: 7.02% +/-0.1229 (p=0.5727)  F: -12.08% +/- 0.1535(p=0.4377) | M: 21.11%+/-0.1394(p=0.1412)  F:-20.796 %+/-0.0916 (p=0.0317)* | M:-2.891%+/-0.0988(p=0.7713)  F:-13.265%+/-.085 (p=0.1296) |
| Open Field | Distance (cm) | M:-13.672% +/- 0.1087 (p=0.2186)  F: -2.575% +/- 0.1231 (p=0.8359) | M:-13.734% +/-0.0951(p=0.1597)  F:8.564 % +/- 0.1401(p=0.5463) | M:-5.235% +/- 0.1141(p=0.6500)  F:17.366% +/- 0.1410(p=0.2292) | M:16.428% +/- 0.0928(p=0.0875)  **F:74.363% +/- 0.1762 (p=0.0003)*** |
| Open Field | Perimeter time (sec) | M:-2.656%+/-0.0340 (p=0.4419)  F:-3.789 +/- 0.0215(p=0.0883) | M:-0.907%+/-0.0363 (p=0.8044)  F:-5.708%+/-0.0347 (p=0.1125) | M:-1.907% +/-0.0336 (p=0.5749)  F:-2.431 % +/-0.0324 (p=0.4598) | **M:-11.36%+/-0.0370 (p=0.0047)***  **F:-7.424 % +/-0.0351 (p=0.0450)*** |

**Supplementary Table 9: Summary of behavioral results at the Jackson Research Lab**

Unpaired Two-tailed t test run on each sex at each time point and genotype. Open field distance is using the total distance traveled in 60 minutes by each mouse/sex/genotype. *****Statistically Significant
